# Efficacy of a gamified digital therapy for speech production in people with chronic aphasia (iTalkBetter): behavioural and imaging outcomes of a phase II item-randomised clinical trial

**DOI:** 10.1016/j.eclinm.2024.102483

**Published:** 2024-02-21

**Authors:** Emily Upton, Catherine Doogan, Victoria Fleming, Pedro Quijada Leyton, David Barbera, Peter Zeidman, Tom Hope, William Latham, Henry Coley-Fisher, Cathy Price, Jennifer Crinion, Alex Leff

**Affiliations:** a UCL Queen Square Institute of Neurology, University College London, UK; b Institute of Cognitive Neuroscience, University College London, UK; c Department of Psychology and Language Sciences, University College London, UK; d St George’s, University of London, UK; e Wellcome Centre for Human Neuroimaging, University College London, UK; f Department of Psychology and Social Science, John Cabot University, Rome, Italy; g Department of Computing, Goldsmiths, University of London, UK; h The Information Lab, UK; i University College London Hospitals NHS Trust, UK

**Keywords:** Aphasia, Speech production, Computerised therapy, Structural MRI, Functional MRI, Randomised controlled trial

## Abstract

**Background:**

Aphasia is among the most debilitating of symptoms affecting stroke survivors. Speech and language therapy (SLT) is effective, but many hours of practice are required to make clinically meaningful gains. One solution to this ‘dosage’ problem is to automate therapeutic approaches via self-supporting apps so people with aphasia (PWA) can amass practice as it suits them. However, response to therapy is variable and no clinical trial has yet identified the key brain regions required to engage with word-retrieval therapy.

**Methods:**

Between Sep 7, 2020 and Mar 1, 2022 at University College London in the UK, we carried out a phase II, item-randomised clinical trial in 27 PWA using a novel, self-led app, ‘iTalkBetter’, which utilises confrontation naming therapy. Unlike previously reported apps, it has a real-time utterance verification system that drives its adaptive therapy algorithm. Therapy items were individually randomised to provide balanced lists of ‘trained’ and ‘untrained’ items matched on key psycholinguistic variables and baseline performance. PWA practised with iTalkBetter over a 6-week therapy block. Structural and functional MRI data were collected to identify therapy-related changes in brain states. A repeated-measures design was employed. The trial was registered at ClinicalTrials.gov (NCT04566081).

**Findings:**

iTalkBetter significantly improved naming ability by 13% for trained items compared with no change for untrained items, an average increase of 29 words (SD = 26) per person; beneficial effects persisted at three months. PWA’s propositional speech also significantly improved. iTalkBetter use was associated with brain volume increases in right auditory and left anterior prefrontal cortices. Task-based fMRI identified dose-related activity in the right temporoparietal junction.

**Interpretation:**

Our findings suggested that iTalkBetter significantly improves PWAs’ naming ability on trained items. The effect size is similar to a previous RCT of computerised therapy, but this is the first study to show transfer to a naturalistic speaking task. iTalkBetter usage and dose caused observable changes in brain structure and function to key parts of the surviving language perception, production and control networks. iTalkBetter is being rolled-out as an app for all PWA and anomia: https://www.ucl.ac.uk/icn/research/research-groups/neurotherapeutics/projects/digital-interventions-neuro-rehabilitation-0 so that they can increase their dosage of practice-based SLT.

**Funding:**

10.13039/501100000272National Institute for Health and Care Research, Wellcome Centre for Human Neuroimaging.

**Research in context**

**Evidence before this study**

We searched PubMed and Google Scholar from Jan 1, 2015 to Mar 31, 2023, for meta-analyses in English evaluating the dose and intensity of Speech and Language Therapy (SLT) provision for People With Aphasia (PWA). Two key points emerge: 1) most health care systems massively under dose PWA in terms of the hours of SLT that they are provided with; 2) SLT support is almost always focused on the post-acute phase, giving the wrong impression that PWA cannot make meaningful gains after this. The gold-standard remains SLT delivered therapy, but one solution to address these two points is to provide impairment-based therapies via adaptive apps. A key challenge for apps that target speech production is having an automated system that can interpret aphasic speech.

**Added value of this study**

This study establishes that truly self-supporting confrontation naming apps (with software that can manage aphasic speech responses) can help PWA make large gains in their ability to retrieve trained words. These gains translate to real-word usage as PWA were able to describe complex scenes producing significantly more information carrying words. Longitudinal brain imaging identified brain regions related to language perception, production and control that either increase in volume or in task-related activity after practice with iTalkBetter.

**Implications of all the available evidence**

Our findings suggested that PWA should be given access to naming apps that can provide cued retrieval on a trial-by-trial basis. Therapeutic effects are substantial but item specific so the practice items need to be relevant to, and chosen by, the PWA. Two key brain areas in vascular territories spared by most aphasic strokes, the left anterior prefrontal cortex and right-sided primary and secondary auditory areas, likely support these therapeutic effects. Self-led apps are cost-efficient and encourage self-management; properly designed and tested, they can help close the research-practice gap in aphasia rehabilitation. iTalkBetter is being rolled-out for PWA to download and use.

## Introduction

A landmark Canadian study in over 66,000 people dependent on others for their care correlated quality of life scores with the presence of 75 different medical conditions. Aphasia, a disorder of language commonly caused by a stroke, was the condition associated with the lowest quality of life.^1^ Anomia, the inability to retrieve words, is the most frequently reported and frustrating symptom according to people with aphasia (PWA).^2^ While it commonly persists into the chronic phase (more than 6 months post-stroke), and constitutes a serious barrier to communication and functioning in daily life,^3^ there is good evidence that practice-based interventions improve anomia.^4^ Frustratingly, the doses (total hours) of speech and language therapy (SLT) that most PWA receive is far below than that required to provide clinically meaningful improvements in communicative ability.^5^^,^^6^ This under-dosing is a world-wide phenomenon.^7^

One method for increasing the dose of impairment-based SLT is to create automated, self-led digital therapy apps that PWA can use at home without the need for clinical input, providing a cost-effective and convenient approach. Automating standard clinical approaches to improve anomia, such as confrontation naming, is a logical step and a handful of studies have demonstrated the effectiveness of computerised self-led therapies in improving word retrieval deficits.^8^ A major challenge for truly self-led and adaptive anomia digital therapy is finding a way to automate therapy progression by providing trial-by-trial feedback. This requires software that can accurately classify aphasic speech responses in real time so that incorrect naming attempts are identified and correct naming attempts lead to increasingly harder items being presented. iTalkBetter utilises custom software (NUVA: naming utterance verifier for aphasia) that can do this with an accuracy comparable with practicing SLTs,^9^ thus allowing PWA to amass many hours of individualised therapy practice without the need for SLT input.

We wished to test the efficacy of iTalkBetter within the confines of a randomised clinical trial. As the effectiveness of anomia therapy using confrontation naming is so well established and is known to be predominately item-specific,^10^ we chose to randomise therapy items (words) across PWA rather than randomising PWA to iTalkBetter or not; although we did include an extra within-subject control block of ‘no therapy’. Within-subject designs are more powerful than between-subject designs as the variability in post-stroke function varies dramatically across patients.^11^ We carried out the by-item randomisation by carefully matching pairs of words based on both their key psycholinguistic variables and individual participants’ accuracy over two pre-therapy baseline testing sessions (see Supplementary material). We then randomly allocated one item of the pair to the training corpus while the other acted as the untrained control. In order to test whether any gains in confrontation naming carried-over into communicative ability, we also created bespoke composite pictures containing both trained and untrained items for participants to describe.

**Fig. 1:**
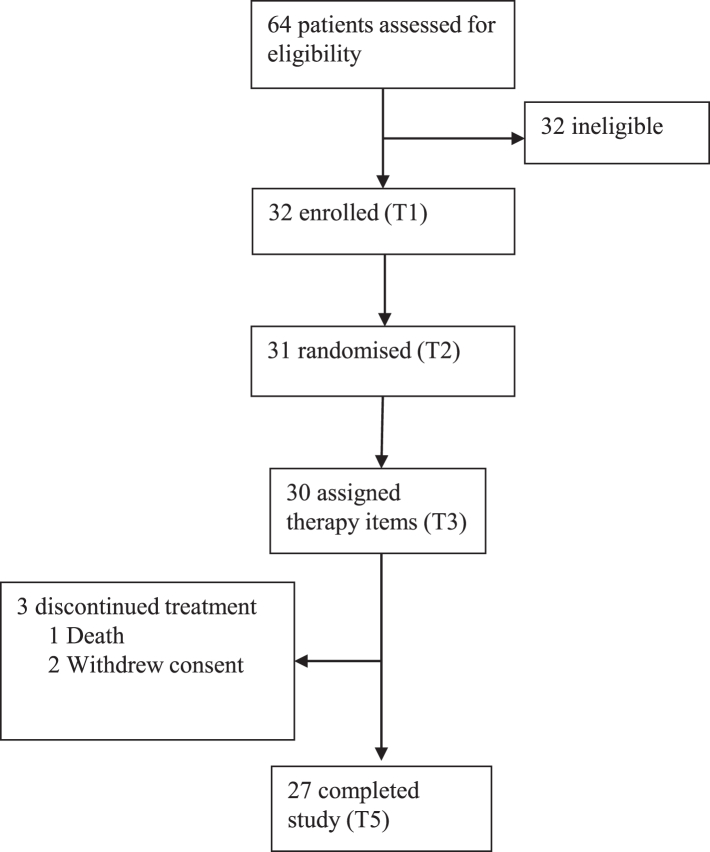
**Consort diagram**.

PWAs’ response to a given dose of mass-practice based therapy is notoriously variable. One key factor is the location and extent of damage to brain regions and networks that support language recovery.^12^ As these regions are not yet known for speech production, we planned serial measurements of brain structure using MRI. Thirty-two PWA were recruited for the study and data is presented for the 27 participants who completed all testing time-points (see Fig. 1). The literature regarding therapy-induced changes in brain function supporting confrontation naming is somewhat mixed. Some studies have reported correlations between improved naming ability and task-induced regional activity in left-hemisphere, perilesional regions,^13^ while others report more widespread activation throughout the left hemisphere language networks^14^; the role of the right hemisphere has also been highlighted.^15^^,^^16^ As well as individual variability playing a role, it is likely that both the composition of the practice-based therapy and the in-scanner tasks used to probe recovery of language function help explain these disparate findings.^17^ With this in mind, we employed a longitudinal design with serial measurements of brain structure and language function using structural MRI (sMRI) and task-based functional MRI (fMRI) either side of two six-week blocks, the first a control block with no therapy, the second the iTalkBetter therapy block.

## Methods

### Study design/Ethics

Using co-design principles, we created a novel, computerised, self-led therapy (‘iTalkBetter’) with the aim of improving word retrieval impairments in PWA using a high-dose treatment approach. iTalkBetter involves massed practice of single word picture naming, supported by error-reducing, phonological cueing. In line with previous research, we hypothesised that iTalkBetter would improve the production of trained items compared with matched, untrained items.^18^ Untrained items controlled for any confounding effects related to the main outcome measure, such as test-retest effects that might be seen across its five repetitions (i.e., improved naming scores caused by increasing familiarity with the outcome test rather than genuine, practice-based improvement due to the training).

**Fig. 2:**
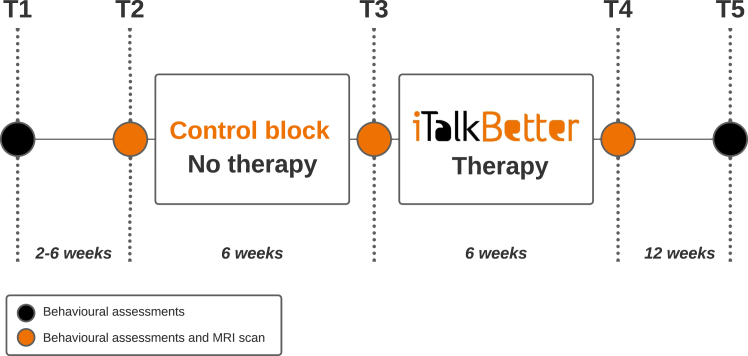
**iTalkbetter study design**.

We used a repeated measures design with five testing time points (T1–T5), each 2–12 weeks apart (Fig. 2). The therapy block (T3–T4) consisted of six weeks of self-managed, app-based therapy (iTalkBetter), with a target dose of 60 h (∼90 min/day). Outside of the therapy block, participants had no access to iTalkBetter therapy. Ethical approval for the iTalkBetter study was granted by the National Research Ethics Service Committee East of England, Cambridge (18/EE/228) and the trial was registered with ClinicalTrials.gov ID: NCT04566081.

#### Participants

Participants were recruited from the Predicting Language Outcome and Recovery After Stroke (PLORAS) database (Wellcome Centre for Human Neuroimaging, UCL^19^) and from a local outpatient clinic (JC’s NHS aphasia clinic). The inclusion criteria were: (i) chronic post-stroke aphasia (at least six months post-stroke); (ii) adults, aged 18 or over; (iii) English as a dominant language; (iv) anomia in the absence of a severe speech output deficit as evidenced by: (a) impaired word retrieval on the ‘Object naming’ subtest of the Comprehensive Aphasia Test (CAT) (score > 12/24); and (b) reasonably intact single word repetition on the ‘Repetition’ subtest of the CAT (score > 12/24). Exclusion criteria were: (i) diagnosis of developmental language disorders; (ii) major co-existing neurological or psychiatric disorders; (iii) unable to give informed consent.

Participants had a mean age of 62 years (SD = 12 years) and a mean time since stroke of 83 months (SD = 67 months). We obtained written informed consent from all PWA to take part in the study.

#### iTalkBetter therapy

The iTalkBetter app was developed for the study in collaboration with a software development company (SoftV) and PWA and their carers. The therapy is a single word picture-naming task with error-reducing, phonological cues, and includes 220 lexical items from all major word classes. The therapy content also includes both concrete (highly imageable words which portray tangible concepts e.g., ‘cat’) and abstract words (low imageability words which refer to intangible concepts e.g., ‘confidence’).

A novel automatic speech recogniser was developed by a member of the team and incorporated into the therapy.^9^ This naming utterance verification system (NUVA) utilised a deep learning element that classified, in real time, a naming attempt as correct or incorrect. This allowed the app to determine the next cue level and also enabled the provision of immediate feedback to the PWA for each individual practice of a word (trial). See Supplementary materials for the item progression algorithm.

iTalkBetter also utilises gamification to reduce the boredom and fatigue effects often associated with repetitive and intensive tasks, thereby decreasing participant drop-out rates.^20^ In iTalkBetter, bright and engaging colours are used in the therapy via the use of an ‘outer space’ theme, which was designed and developed by our software team, SoftV. Visual feedback, in the form of words (‘Well done!’) and actions (pictures are collected and stored in sight on screen), are also provided to motivate users when items are correctly named.

#### Primary outcome: word retrieval test (WRT)

The Word Retrieval Test (WRT) is a novel assessment developed for the iTalkBetter therapy and was used as the primary outcome measure for the study. It is a single word picture naming assessment that was completed at all time-points (T1–T5). The WRT was created with two main requirements: inclusion of a large number of lexical items to be sensitive in capturing change, and inclusion of trained and untrained items to assess whether treatment effects were item-specific or if they generalised to untrained items. The WRT was shown to have high concurrent validity with the ‘Object naming’ subtest of the CAT, r = 0.733, *p* ≤ 0.001, suggesting the test is a valid measure of single word naming.

The test consisted of 220 lexical items (nouns, adjectives, verbs and pronouns) which were chosen from the initial therapy content to be representative of the entire initial corpus based on the following variables: word class, syllable length, concreteness,^21^ frequency^22^ and age of acquisition.^23^ The pictures in the WRT, however, were different to those in the therapy to avoid participants’ rote learning the association between the spoken word and the picture (identity priming).^24^

#### Randomisation of the therapy content

The therapy content consisted of 220 words. Half (n = 110) were ‘core therapy’ items of very high frequency and were the same for all participants, but did not form part of the WRT. The other 110 were ‘trained’ items, a list that varied across participants. These words were tested in the WRT alongside 110 matched, ‘untrained’ items which were not presented in therapy. Participants were tested at the two pre-therapy time points on all 220 words and scored for accuracy (0—incorrect; 1—correct). Baseline performance on the WRT at T1 and T2 was then used to assign words within a pair to either trained or untrained word lists by balancing performance between pairs for each participant.

We used treatment allocation by minimisation as outlined by Altman and Bland in order to allocate items to each participant.^25^ The random number generator in excel was used to select which item in the first word pair was assigned to the ‘trained’ word list (word given the highest random number) and subsequent word pairs were allocated by hand to balance accuracy scores across the trained and untrained word lists. Any time that this count was equal (equipoise), the random number generator was employed again (Supplementary Table S2). The 110 words which were selected to be untrained were removed from the therapy content and the 110 trained words were kept in the therapy content. This allocation method created unique lists of trained and untrained items for each participant which were matched for both key psycholinguistic variables and baseline performance.

iTalkBetter therapy content included abstract words, therefore the WRT had to be devised so that it was also reliable at measuring improvements in the retrieval of this word class. A challenge for abstract word elicitation is participants’ agreement of the word-picture pairings (i.e., the target spoken word for a particular picture), which is separate from the ability to retrieve the word. To ameliorate this issue, two distinct components were incorporated into the WRT: a ‘fly by’ in which participants saw and heard the target names of 20 pictures (no response required); and, a ‘naming test’ in which participants saw the same 20 pictures again (in randomised order) and had to free-name the pictures. To ensure consistency in exposure to items, all 220 words (both trained and untrained; abstract and concrete) were included in both components. The fly-by necessarily imposed a working memory component on the task, but it was deemed necessary in order to fairly test the effects of therapy on both abstract and concrete words. As trained and untrained items were treated equally in the fly-by, any gains on trained items alone would most likely be due to improvements in a PWA’s word retrieval ability rather than their short-term memory skills. The WRT was presented within the iTalkBetter therapy app, which audio recorded participants’ responses. Following the test, responses were scored by hand.

#### Secondary outcome: spoken picture description (SPD)

A novel Spoken Picture Description task was used as a secondary outcome measure and was completed at T2, T3 and T4, and included both trained and untrained items (Supplementary Fig. S3). This assessment was created to investigate whether any gains seen at the single word level (in the WRT) generalised to another, more ecologically valid, speech production task.

The SPD consisted of two pictures of composite scenes that depicted a selection of trained and untrained words from the WRT to enable the direct comparison of naming performance between the two assessments. Items from the WRT were selected only if both words in the matched pairs could be included. As one word in a pair was always trained and the other was always untrained, this ensured an equal number of trained and untrained words from the WRT were incorporated into the SPD for each participant. The scenes also included trained items from the core therapy content that were not tested in the WRT, and items that were not trained or tested in the iTalkBetter study.

Each SPD was presented digitally as a PDF. Participants were given 2 min to describe what was happening in each picture and were instructed to try to use full sentences. Responses were audio recorded and then transcribed following the assessment sessions. The transcriptions were analysed by counting the number of appropriate: WRT words (trained and untrained words); core therapy words (trained in the therapy but not tested in the WRT); and untrained and untested words (words not trained in the therapy or tested in the WRT). The number of unique information carrying words were counted for each word type, any repetitions of words were not included in the analysis.

Due to the COVID-19 pandemic we had to pause the start of the study to September 2020. Seven of the participants’ testing sessions were completed entirely remotely via the video calling platform, Zoom (https://zoom.us/). For the remaining 20 participants, testing sessions were either completed via Zoom, in-person at University College London, or in participants’ homes. The trial closed March 2022.

#### Baseline data

Baseline behavioural and key demographic data are presented in the Supplementary Table S1. All participants were impaired on the CAT in both expressive and receptive subtests; however, all had poorer single word naming abilities in comparison to spoken single word comprehension, and, although variable, no participant had a severe impairment in single, real-word repetition (i.e., a score of less than 12/24).

#### MRI scanning and analysis pipeline

Participants who were able and available for MRI scanning (dependent on safety requirements and the COVID-19 pandemic), completed structural and task-based functional scans at T2, T3 and T4 (each six weeks apart).

#### sMRI acquisition, lesion identification and longitudinal voxel-based morphometry

For sMRI acquisition, a T1-weighted 3D modified driven equilibrium Fourier transform sequence was used, which produced 176 contiguous sagittal slices with a 256 × 224 matrix (resolution = 1 mmᶟ; repetition time/echo time/inversion time = 7.92/2.48/910 ms; flip angle = 16°). To identify PWA’s lesions in the present study, and for preprocessing prior to statistical analysis, the Automated Lesion Identification (ALI) toolbox in SPM12 was used.^26^

To investigate therapy-induced change in brain structure, the same processing pipeline employed in a previous aphasia intervention study was used.^27^ Change in the volume of grey matter (GM) and white matter (WM) for each participant over the therapy and pre-therapy blocks, separately, was calculated using serial longitudinal registration. The pre-therapy probabilistic change maps were then subtracted from the therapy change maps to produce two final images for each participant which represented change in voxel volume over the therapy block, over and above change in the pre-therapy block, for GM and WM, respectively. The images were normalized into MNI space and smoothed with an isotropic kernel of 8 mm full width at half maximum (FWHM).

The images were entered into three separate simple linear regression models to investigate the following changes in brain volume effects at a group level: (1) as a simple main effect of practicing with iTalkBetter (a simple comparison with the pre-therapy block where there was no practice); (2) a parametric relationship with response to therapy (percentage improvement); (3) a parametric relationship with the dose of therapy (hours completed). For Analyses 2 and 3, item-exposure was entered as a covariate of no interest due to differences in exposure to trained items across participants. The within-subject pre-processing design accounted for between-subject effects so no further regressors were included. The statistical voxel-level threshold was set at *p* < 0.001 uncorrected, and to correct for multiple comparisons, significant clusters are reported at the family-wise error (FWE) *p* < 0.05 threshold.

#### fMRI acquisition

For the task-based fMRI acquisition, a gradient echo planar imaging (EPI) sequence was used (matrix size = 64 × 64; repetition time/echo time = 3080/30 ms; flip angle = 90°; field of view = 192 × 192; slice thickness = 2 mm, inter-slice gap = 1 mm). Each task-based functional run consisted of 66 volumes per time series. This included five ‘dummy scans’ to allow for magnetisation to reach equilibrium.

The task-based fMRI experiment followed an existing protocol as the data was collected as part of the PLORAS research project.^28^ The experiment was a block design and consisted of five tasks: two finger press response tasks (semantic picture decision and semantic auditory decision) and three overt speech tasks (object naming, verb production and sentence production). Each task had 20 pictures or auditory stimuli which were made up of two items, either people, animals or objects, with names of one to four syllables.

Each task was presented in a separate scan run and the 20 items in a task were presented in four blocks of five, at a rate of one every 5 s. Each block lasted for 20 s to allow for sufficient time for the BOLD response to peak. At the end of a block was a 16-s rest block to allow activation, and the proportion of oxygenated and deoxygenated blood, to return to baseline. During these breaks, a fixation cross was displayed on the screen. Before the next block started, written instructions (for example, ‘Name the verb’) were given.

Picture stimuli were displayed for 2.5 s (followed by a 3.5 s fixation cross) on a projector that participants viewed via an adjustable mirror on the head coil. Auditory stimuli were presented through MRI compatible headphones (MR Confon, Magdeburg, Germany) for 1.75–2.5 s, during which, a fixation cross was shown. Speech responses were recorded via a noise-cancelling MRI microphone (FOMRI IIITM Optoacoustics) and were also transcribed manually online. In the semantic decision tasks, an MRI compatible button box was used to record responses (participants used two fingers on the same hand to press one of two buttons).

The audio recordings were used to verify the online transcribed speech responses and the button box responses were received from the recorded data. Trials were marked as either correct, incorrect, or no response.

#### fMRI pre-processing and 1st and 2nd level analysis

In the scanner, measures were taken to minimise head movement, for example, using padding around the participant’s head, but a small amount of movement was unavoidable, particularly for speech production tasks. As a result, motion correction was necessary to ensure the location of a single voxel within the field of view was constant, and any changes in signal intensity were due to a change in the BOLD response. To correct for movement in the pre-processing steps, functional images were spatially realigned to the first EPI volume, to bring all of the data into the same orientation, and un-warped to account for residual movement related variance induced by susceptibility-by-movement interactions, using the unwarp toolbox in SPM12.

Following motion correction, the structural T1 image was co-registered to the mean EPI image and spatially normalised into standard MNI space using the unified normalisation-segmentation tool in SPM12 using the deformation parameters.

In the first level design matrix, all five of the task-based functional pre-processed images from the three time points (T2, T3 and T4) for each individual were entered into a fixed-effect general linear model (GLM).^29^ For each of the five tasks (two non-verbal button box tasks and three speech tasks), the stimulus onset times were modelled as single, spike events, alongside response durations and reaction times, with four distinct regressors for each task: 1) instructions; 2) correct responses; 3) incorrect responses; and 4) no responses. However, due to the severity of aphasia for some participants in the study, for the three speech tasks distinctions were not made between ‘correct’ and ‘incorrect’ responses. Instead, these two response types were collapsed together and coded as ‘speech responses’ to enable all participants to be included in the analysis. Stimulus functions were then convolved with a canonical haemodynamic response function. To remove low frequency confounds (noise) caused by ‘scanner drift’ (linear changes over time in signal intensity), temporal filtering was performed using a high-pass filter of a set of discrete cosine basis functions with a cut-off of 128s.

To investigate functional changes from pre-therapy to post-therapy at the group level, two contrast images were produced from the first level analysis. The two contrast images represented change in activation over pre-therapy (T2–T3, modelled as −1.1) and change over therapy (T3–T4, modelled as −1.1) for the three speech production tasks. To investigate changes in activation from pre-therapy to therapy at the group level, the contrast images for all participants were entered into three separate paired sample t-tests in SPM12, examining both increases and decreases in activation: (1) as a simple main effect of practicing with iTalkBetter; (2) a parametric relationship with response to therapy (percentage improvement); (3) a parametric relationship with the dose of therapy (hours completed). For Analyses 2 and 3, item-exposure was entered as a covariate of no interest due to differences in exposure to trained items across participants.

As with the structural analysis, the statistical peak threshold was set at *p* < 0.001 uncorrected, and significant clusters are reported at *p* < 0.05 using family-wise error (FWE) correction to control for multiple comparisons across the whole brain using random field theory.^30^

**Fig. 3:**
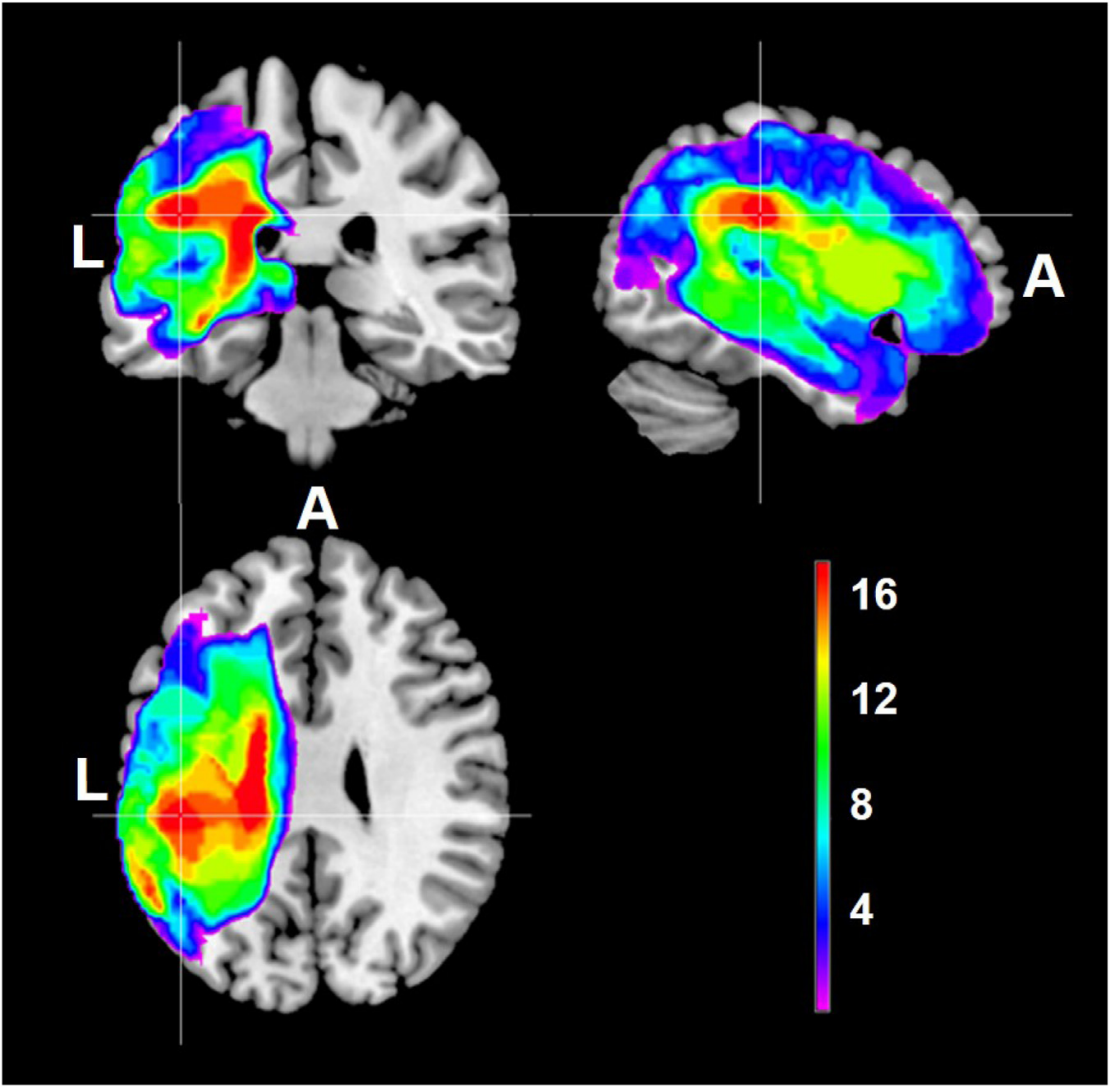
**Lesion overlap map for 17 participants in Montreal Neurological Institute (MNI) space.** A = anterior, L = left. The colour bar represents the number of participants with a lesion in a voxel, from 1 (purple) to 17 (red).

Fig. 3 displays the lesion distribution for the 17 participants who were able to complete all three scanning sessions. All had left hemisphere strokes, with most participants having extensive and widespread damage, encompassing the temporal, parietal and frontal lobes. Maximal overlap was in the white matter tracts of the corona radiata and the corpus callosum, and in the grey matter of the supramarginal gyrus.

#### Behavioural outcomes

To investigate the efficacy of iTalkBetter, raw percentage change over the therapy block (T3–T4) was compared with raw percentage change over the no therapy block (T2–T3), for both primary and secondary outcome measures. Repeated measures analysis of variances (ANOVAs) were performed using two within-subject factors each with two levels: block (no therapy (T2–T3) vs. therapy (T3–T4)); and item (trained vs. untrained lexical items).

Post-hoc, paired-sample, two-tailed t-tests explored significant interactions and examined change over the baseline (T1–T2) and maintenance (T4–T5) blocks. The alpha-level for all analyses was *p* < 0.05. We report both unstandardized effects (% change) and standardised effects (Cohen’s *d*), the latter using the conservative between group method rather than a version of the repeated-measures Cohen’s *d*.

### Role of the funding source

The funder of the study had no role in study design, data collection, data analysis, data interpretation, or writing of the report. All authors had access to the dataset and AL had final responsibility for the decision to submit for publication.

## Results

### iTalkBetter dose

On average, participants spent 45 h (SD = 16) on iTalkBetter therapy over 6 weeks, completing 17,538 (SD = 6262) individual trials. Participants completed the error-reducing cueing paradigm on average 27 times (SD = 17) for each lexical item.

### Therapy effects: word retrieval test

**Fig. 4:**
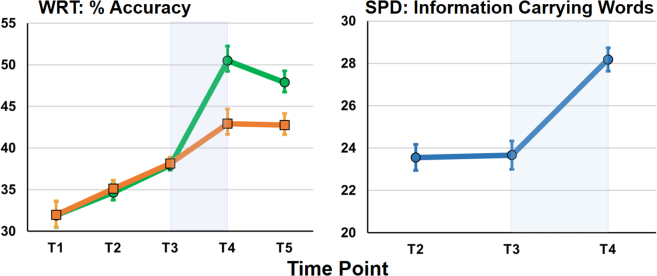
**(Left) performance (%) on the Word Retrieval test (WRT) at the five testing time-points.** The green line denotes trained items and the orange line matched, untrained items. The blue shaded area covers the 6-week therapy block. (Right) Number of information carrying words produced on the Spoken Picture Description task at T2, T3, and T4. Error bars are within-subject standard error of the mean.

There was a significant interaction between block (therapy vs. no therapy) and item (trained items vs. untrained items) (*F* (1,26) = 10.41, *p* = 0.003) (Fig. 4), which was driven by improvements for trained items over the therapy block (*t* (26) = 4.04, *p* ≤ 0.001). The effect size was moderate to large as indicated by unstandardised and standardised measures: 13% (SD = 11.7) (absolute change), Cohen’s *d* (0.52). In terms of the number of lexical items improved by iTalkBetter, this equates to 220∗0.13 = 29 words (SD = 26) as the therapy corpus consisted of 110 trained words (unique to each participant and tested in the WRT) and 110 core words (common to all participants, but not tested for). Both trained and core words had equal exposure rates in the iTalkBetter therapy protocol.

There was a small but significant improvement on the WRT from T1 to T2 (baseline change) (2.95%, *t* (26) = 2.91, *p* = 0.007) across all items (to-be-trained and untrained); however, there were no significant difference between to-be-trained and untrained items either between T1 to T2; and T2 to T3 (*t* (26) = 0.13, *p* = 0.9), indicating this was a stable improvement over the three pre-therapy time points, likely due to learning or familiarity effects from the ‘fly-by’ component of the test.

Over the maintenance block (T4–T5) there were no significant changes in performance accuracy (trained items: *t* (26) = 1, *p* = 0.33; untrained items: *t* (26) = 0.13, *p* = 0.9) suggesting that task familiarity had saturated.

### Therapy effects: spoken picture description

No significant interactions were found between block (therapy vs. no therapy) and item (trained items vs. untrained items), but there was a main effect of block (*F* (1, 26) = 4.82, *p* = 0.04). Post-hoc analyses (a paired sample t-test and a Wilcoxon signed ranks test) examined change in the number of unique information carrying words produced, as well as change in the number of trained items (both core therapy items and trained items tested in the WRT). Significant increases were found in the retrieval of information carrying words over therapy in comparison to no therapy (T2–T3), *t* (26) = 2.2, *p* = 0.04. Average effect size was +4.4 words, Cohen’s *d* (0.42).

### Structural brain changes

**Fig. 5:**
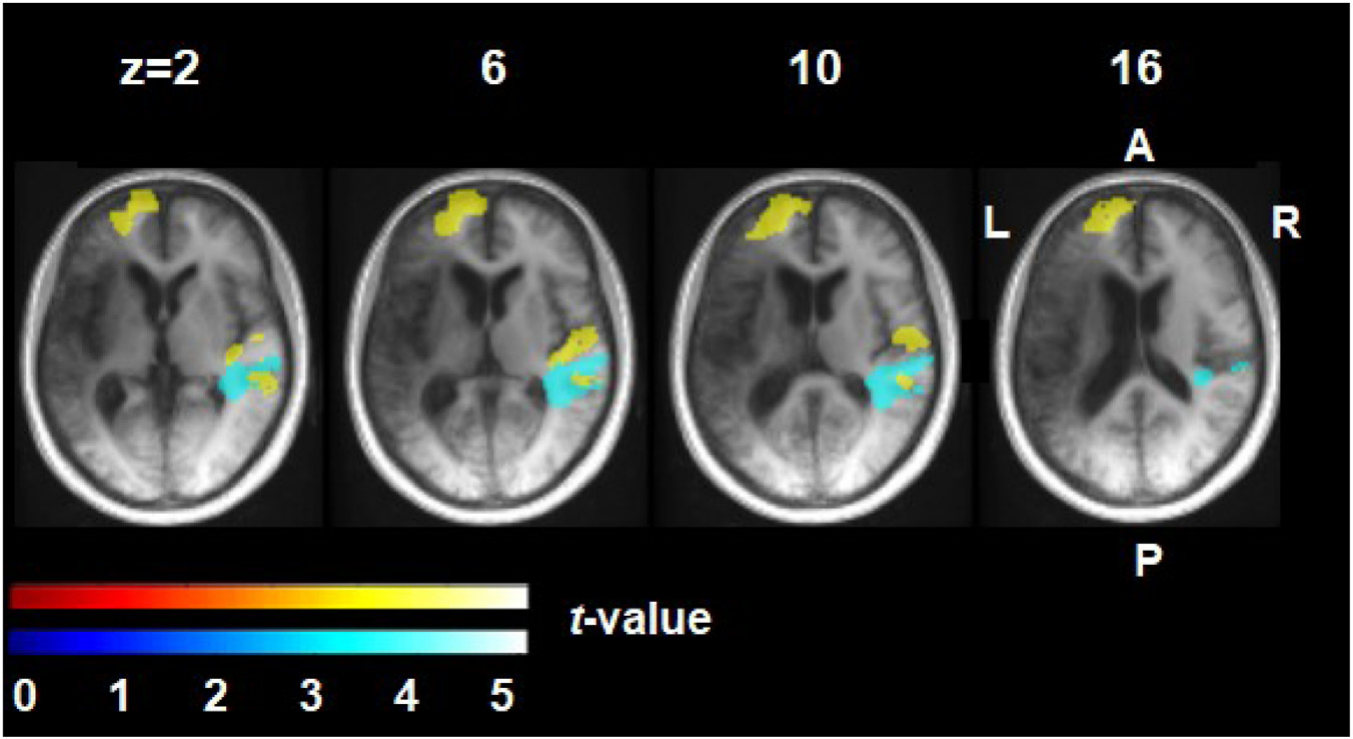
**Significant brain clusters with increased volume post-therapy.** The brain template is the average T1-weighted MRI image for the 17 participants at T2. A = anterior; P = posterior; L = left; R = right. Yellow = grey matter and blue = white matter. Numbers are MNI coordinates.

Three significant clusters which increased in volume as a general effect of partaking in the therapy programme were found, compared with the control block of no practice (Fig. 4, Fig. 5, Table 1). These were: in grey matter in the left anterior prefrontal cortex (BA10) and in the right planum temporale, the homologue region to Wernicke’s area (BA21 and BA22); and in the white matter tracts underlying BA22 and BA21 in the right hemisphere. No significant peaks or clusters were found to correlate with therapy dose or response to therapy.

**Table 1:** Significant clusters showing increased brain volume over the therapy block compared with the no therapy block.

Structural brain changes					
Region	*k*	*T*	*x*	*y*	*z*
Grey matter					
Left anterior prefrontal cortex	1356	5.68	−8	62	22
		5.54	−14	72	2
		5.24	−6	66	12
Right planum temporale	957	5.92	56	−30	8
Middle temporal gyrus		5.66	62	−36	2
Planum temporale		5.35	50	−34	4
White matter					
Right planum temporale	982	5.05	60	−26	4
Middle temporal gyrus		4.82	62	−40	6
Planum temporale		4.65	40	−46	8

The co-ordinates represent MNI coordinates of the first three peak voxels within each cluster; ‘k’ is cluster size; and ‘T’ the t-value.

### Functional brain changes

**Fig. 6:**
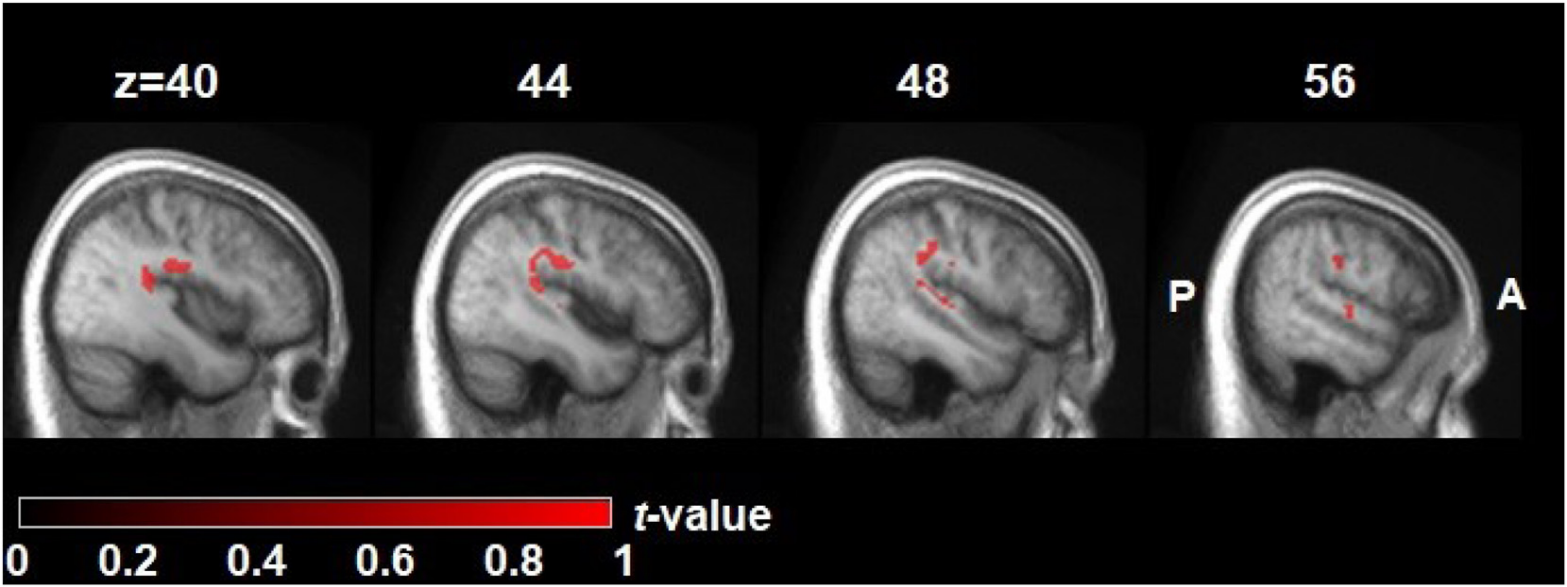
**Significant right hemisphere cluster with higher fMRI task-related activity (within-subject contrast = change over therapy block—change over no therapy block) correlated with dose (between subject variable).** The brain template as per Fig. 5. A = anterior; P = posterior. The colour bar represents t-values at that voxel. T values and MNI coordinates shown.

Regarding the in-scanner performance during the three speech production tasks, a repeated measures ANOVA (one factor: time; three levels: T2, T3 and T4), found no significant difference in the number of overall speech responses given for each of the three tasks over the three time points, F (2,15) = 1.5, *p* = 0.24 (the fMRI stimuli were untrained items). Regarding the group-level analysis of fMRI data, a single significant cluster was identified which increased in activation over the therapy block in comparison to the no therapy block, and was correlated with dose of therapy (hours completed). This cluster was in the right temporo-parietal region and comprised of 176 contiguous voxels (pFWE-corr = 0.007). The first three subpeaks were in the posterior parietal operculum, supramarginal gyrus and Heschl’s gyrus (primary auditory cortex), see Fig. 6 and Table 2.

**Table 2:** First three subpeaks in the only cluster of fMRI task-related activity that survived whole brain correction for multiple comparisons.

Region	*T*	*x*	*y*	*z*
Right Posterior Parietal Operculum	6.66	42	−22	26
Right Supramarginal gyrus	4.87	57	−19	26
Right Heschl’s gyrus (1⁰ Aud cortex)	4.80	36	−34	17

Data labels are taken from the automated anatomical parcellation AAL3 atlas.

## Discussion

Six weeks of practice with iTalkBetter significantly improved PWA’s ability to retrieve words that they had trained on. On average, the participants improved by 13% and these gains were maintained 12 weeks following the cessation of therapy. No similar effects were found for untrained items, supporting our hypothesis that treatment effects would be item specific. These results add to the growing evidence-base that effective aphasia therapy can be delivered digitally.^31^ In terms of effect-size, the present findings align with those from Palmer and colleagues’ Big CACTUS RCT, which utilised the StepByStep computer programme^8^ to target speech production of 100 personally relevant words in PWA over a six-month period (16% improvement on trained items). Unlike StepByStep, iTalkBetter participants were able to independently use the app with minimal or no help from a communication partner or the research team. Importantly, the gains made in naming ability were mirrored by significantly improved retrieval of items in our tailormade spoken picture description task, demonstrating a generalisation of learning effects from the single word naming therapy to a less constrained task.

Over the course of the six-week therapy block, participants completed an average of 45 h of therapy. Although below the clinical trial target of 60 h, this is within the recommended dosage required to improve language functions.^5^ To ensure a high dose of therapy was achieved, the research team provided regular encouragement (through phone calls and emails) to motivate participants who were falling behind, as in previous studies.^8^^,^^32^ Despite this encouragement, six participants achieved less than half of the target dose (<30 h) due to time constraints, illness, fatigue or low motivation. No individual dropped out due to not being able to engage with the treatment programme; however, this type of high dose, high intensity therapy may not be suitable for all PWA. In the roll-out phase of iTalkBetter, PWA will be under less of a time pressure to achieve the target dose. Additionally, although we have shown that 45 h is effective on average, the roll-out will provide us with more of an insight into the optimal delivery of the therapy (dose and intensity) at an individual level.

With regards to the structural brain imaging findings, longitudinal studies in healthy individuals have shown that experience-dependent plasticity can be observed in brain regions in response to training new skills such as juggling, playing golf and foreign language learning; even after only a few weeks of intense practice.^33^ We observed significant increases in grey and white matter volumes in two brain areas associated with practicing iTalkBetter for 6 weeks (compared with the control block of no practice), the right temporal lobe (primary and secondary auditory cortices) and the left anterior prefrontal cortex (BA10). Longitudinal, therapy-based structural imaging studies in PWA are rare; however, changes in grey and white matter in both the left and right hemispheres have been reported previously.^27^^,^^34^ In the right hemisphere, we found structural changes in primary and secondary auditory cortices which supports the proposal that these regions play a special role in matching auditory expectations with spectral-temporal processing from auditory feedback during speech production.^35^ In a similarly conducted, app-delivered therapy for PWA with auditory comprehension deficits, practice-related changes were also seen in the right planum temporale.^27^ These structural changes may reflect the recruitment of regions typically involved in language processing as a result of listening to the large number of auditory cues provided within iTalkBetter; or, they could reflect increased top-down attention to the self-production of trained items. This latter interpretation is consistent with a longitudinal study in PWA that identified increases in right planum temporale volumes associated with improved naming ability.^36^

Unlike the right hemisphere changes, those in the left hemisphere involved a region (BA10) not classically associated with language function. However, BA10 has previously been shown to be one of the left hemisphere regions whose preservation predicts a greater response to anomia therapy.^37^ It has been linked to multiple functions including, but not limited to, cognitive processing, memory, organising behaviour, attention, awareness of competence and inhibition. It is also part of the salience (cingulo-opercular) network where activity changes following an error-reducing phonemic cueing therapy (which was similar to the iTalkBetter therapy programme),^38^ have been associated with both immediate speech improvements and long-term maintenance (three months post-therapy). In another error-reducing phonological cueing therapy study with three PWA, Fridriksson and colleagues^39^ found that bilateral activity in the anterior prefrontal cortex correlated with naming improvements post-treatment for the participant with the smallest lesion. A positive correlation between task-related activity in the salience network and residual language performance has also been observed in a cross-sectional (rather than longitudinal) study.^40^ Given that in our study both temporal and frontal lobe changes in brain structure are associated with exposure to iTalkBetter rather than therapy dose or percentage improvement, we suggest these regions are likely not directly causing behavioural improvements; however, their presence might be required if PWA are to benefit from practice with it.

This is the first task-based fMRI study in PWA to identify dose-related changes in brain function, so comparisons with other studies must be circumspect. Our analysis identified three closely related regions in and around the right temporo-parietal junction that correlated with the amount of time the PWA spent practicing with iTalkBetter. These activations occurred while PWA carried out three speech production tasks with stimuli unrelated to the trained items in iTalkBetter. Changes over the therapy block are unlikely to be due to either the passage of time or test-retest effects (as there was a control block), or due to in-scanner performance as there was no significant effect on number of correct speech responses given for the three tasks across all three time points. Starting with primary areas and moving up through the cortical hierarchy we identified speech production-related activity in Heschl’s gyrus, posterior parietal operculum and supramarginal gyrus. Regarding primary auditory cortex activity, a very similar pattern was observed in right Heschl’s gyrus during speaking tasks in control partcipants and PWA in a study by Lorca-Puls et al.,^41^ which was interpreted as reliance on a region involved in the normal language network to support residual speech production. The second area is a sensory association area, with the peak of the posterior parietal operculum activation in sub-region OP1 as identified by Eickhoff et al. (part of area SII). This region is traditionally considered to be involved in the processing of somatosensory and auditory inputs^42^ but has also been shown to be modulated by nearby temporal regions during normal speech production.^43^ Finally, the right supramarginal gyrus is strongly connected to its left hemisphere homologue and it is possible that this region is involved in supporting these functions when the dominant left supramarginal gyrus is damaged, which is the case in the majority of the PWA in our study (Fig. 2) and would result in the loss one of the core supramodal language areas (involved in visual and auditory language perception and production). Supporting evidence for this hypothesis is bolstered by a study in PWA demonstrating that right supramarginal gyrus fMRI activity was significantly greater for correctly named items compared with items where naming errors of any type were made.^44^

Our study adds three new important pieces of information to the growing literature on the efficacy of therapy apps for PWA. Firstly, we have established that truly self-led confrontation naming apps (with software that can manage aphasic speech responses) can help PWA make large gains in their ability to retrieve words they have been training on. Secondly, this training significantly improves their ability to describe complex scenes using propositional speech. Thirdly, we have identified brain regions related to language perception, production and control that either increase in volume or in task-related activity after practice with iTalkBetter. The first two points suggest that PWA should be given access to naming apps that can provide cued retrieval on a trial-by-trial basis, and that the practice items need to be relevant to, and perhaps chosen by, the PWA. The last point suggests that left anterior prefrontal cortex and right-sided primary and secondary auditory areas likely support these therapeutic effects. Finally, whilst not a substitute for the breadth and complex range of interventions a trained SLT can provide, self-led apps such as ours, which are cost-efficient and promote autonomy and self-management, can help close the research-practice gap in aphasia rehabilitation. iTalkBetter is being rolled-out for PWA to download and use: https://www.ucl.ac.uk/icn/research/research-groups/neurotherapeutics/projects/digital-interventions-neuro-rehabilitation-0.

## Contributors

APL and CP secured the funding for and APL, JC and CD conceptualised the study. The methodology was devised by APL, PQL, DB, PZ, TH, WL, and CP with the investigations carried out by EU, CD, HC-F, PQL, VF and JC. The software was designed by PQL, DB and WL. The data was verified by APL and EU, curated by EU, VF, PQL, and DB with formal analysis by EU, VF, DB, PZ, and TH. APL and CD provided formal supervision. The original draft was written by EU and APL with all authors reviewing, editing and approving the final version. All authors had access to the dataset and AL had final responsibility for the decision to submit for publication.

## Data sharing statement

Anonymised data will be made available for reasonable requests to APL with scientific rational and sound methodology.

## Declaration of interests

None of the authors have any relationships/activities/interests to disclose that are related to the content of this manuscript.

## Appendix ASupplementary data

Supplementary materials

CIP_Phase 2_V2
