# Supplementary material for: Efficacy of a gamified digital therapy for speech production in people with chronic aphasia (iTalkBetter): behavioural and imaging outcomes of a phase II item-randomised clinical trial
Source: eClinicalMedicine. 2024 Feb 21;70:102483. doi: 10.1016/j.eclinm.2024.102483 (PMC11056404; doi:10.1016/j.eclinm.2024.102483)
Supplement: Supplementary materials [file mmc1.pdf]

Supplementary material

Supplementary material 1

1. Baseline behavioural and demographic data 2
2. iTalkBetter App 3
3. Word Retrieval Test: composition and by-item randomisation 4
4. Spoken Picture Description tests 5
5. Sample size calculation (taken from the study protocol) 6
6. Protocol 6
7. References 7

1. Baseline behavioural and demographic data

ID	Gender	Age	Handed-ness	CAT-naming	CAT-repetition	Time since stroke (months)	Type of stroke	Lesion volume (cm^3)
**P1**	M	65	R	29	21	109	Haemorrhagic	158
**P2**	M	58	R	16	16	90	Ischaemic	168
**P3**	M	70	R	13	30	91	Ischaemic	244
**P4**	F	62	R	29	24	22	Ischaemic	52
**P5**	M	64	L	3	23	14	Unknown	232
**P6**	M	59	R	34	29	100	Ischaemic	283
**P7**	M	57	R	28	20	132	Ischaemic	NS
**P8**	F	82	R	27	23	38	Ischaemic	156
**P9**	M	69	L	32	19	136	Ischaemic	NS
**P10**	M	56	R	26	24	156	Ischaemic	NS
**P11**	M	62	R	24	28	149	Ischaemic	NS
**P12**	M	64	R	0	26	62	Ischaemic	NS
**P13**	M	64	R	17	28	74	Ischaemic	NS
**P14**	F	31	L	33	28	60	Haemorrhagic	NS
**P15**	M	68	R	30	19	184	Ischaemic	NS
**P16**	M	64	R	28	22	79	Ischaemic	158
**P17**	F	56	R	25	24	33	Ischaemic	352
**P18**	M	73	R	35	30	43	Ischaemic	109
**P19**	F	61	R	1	13	30	Ischaemic	150
**P20**	M	67	R	26	22	320	Ischaemic	376
**P21**	M	60	R	32	30	43	Ischaemic	166
**P22**	M	70	L	29	27	106	Unknown	264
**P23**	M	64	R	16	19	31	Ischaemic	130
**P24**	F	54	R	29	27	37	Ischaemic	32
**P25**	F	55	R	30	32	19	Ischaemic	191
**P26**	M	45	R	35	32	7	Ischaemic	76
**P27**	M	64	R	6	18	84	Ischaemic	386
Mean		62		23	24	83		194
SD		9		4	5	67		103
Max				48	32			
Cut-off				38	12			

Table S1: baseline behavioural and demographic data for the 27 PWA completing the study. CAT = Comprehensive Aphasia Test,[1] ‘Object naming’ subtest (cut-off <38); and ‘Repetition’ subtest of the CAT (cut-off >12). NS = No Scan

2. iTalkBetter App

iTalkBetter is a digital word retrieval therapy app, which was completed independently at home on a computer tablet. It consists of single word picture naming that works via mass practice using error reducing learning (vanishing cues).

Picture naming paired with error-reducing, vanishing phonological cues has been shown to facilitate word retrieval following therapy with long-term benefit.[2,3] For this reason, vanishing cues were incorporated into the iTalkBetter therapy task (Figure S1). In the task, when participants first saw a picture, they heard the name of the picture (full word cue (FC)). They then had to repeat the picture's name. If the correct name was produced, the participant saw the image again but heard only the initial phoneme (initial phoneme cue (IP)). They again had to say the whole name. If the name was said correctly, the participant saw the picture for a third time but received no auditory cue (no cue (NC)) and once more, they had to say the whole name of the picture.

If at FC a participant said the incorrect name, they were given two further instances to name the picture at FC before the therapy moved on to the next word. If an incorrect name was produced at either IP or NC, the picture was presented again at the previous cue level. In the next therapy cycle, the iTalkBetter therapy presented the picture at the cue level the participant achieved in the previous therapy cycle. For example, if the name was produced correctly at NC in the previous cycle, the picture was first presented at NC; if correct in the previous cycle at IP, the picture was first presented at IP; and if the participant was unable to move beyond FC, the picture was presented at FC.

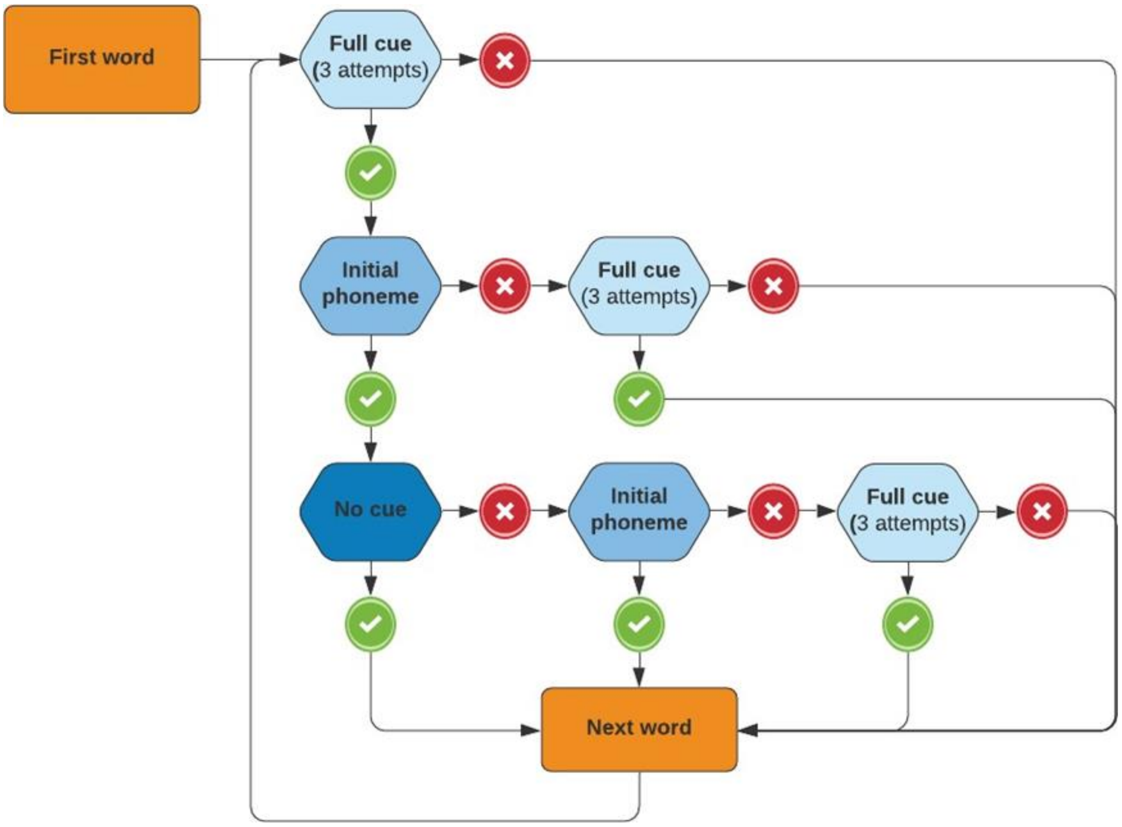

Figure S1: Error-reducing learning.

A novel automatic speech recogniser was developed by a member of the Neurotherapeutics group at UCL and was incorporated into the iTalkBetter therapy.[4] This naming utterance verification system (NUVA) utilised a deep learning element that classified whether the correct or incorrect name was produced in real time. This allowed the app to determine the next cue level and also enabled the provision of immediate feedback on a trial-by-trial basis, thereby improving word retrieval outcomes.[5] If a user produced the correct name, 'Well done!' appeared on the screen and 'Kenny', the spaceman, hoovered up the picture into his 'space backpack'. The space backpack stored pictures on screen, providing a visual representation of correctly named items. If the incorrect name was produced, the picture floated off into 'outer space' (Figure S2).

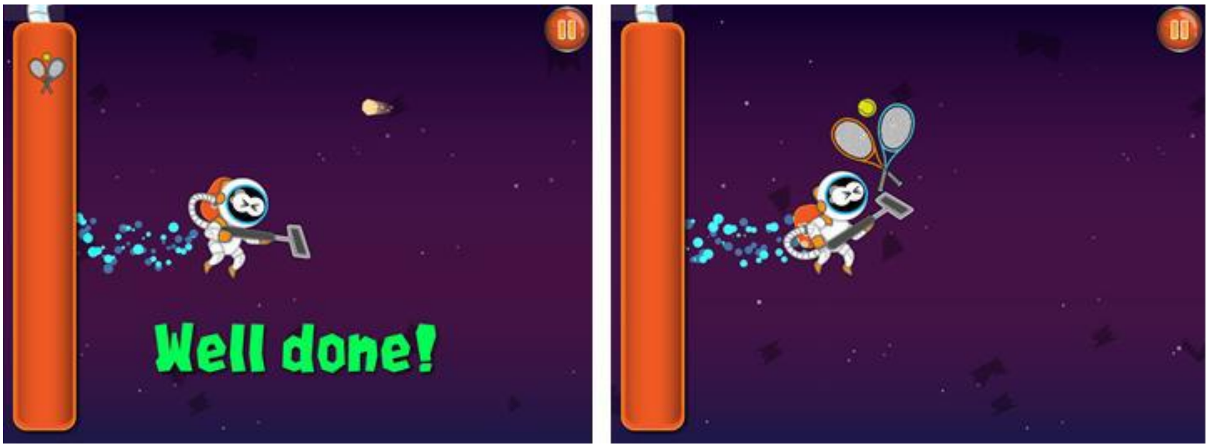

Figure S2: Therapy feedback. Left: feedback for correct response. Right: feedback for incorrect response.

3. Word Retrieval Test: composition and by-item randomisation

Pair		T1	T2		T1	T2
1	bread	1		teeth	1	1
2	girl	1		train	1	1
3	wheel	1	1	juice		1
4	knife	1	1	snow		1
5	chicken	1	1	orange		1
6	upstairs	1	1	downstairs		1
7	soup	1	1	flag	1	
8	gift	1	1	hole	1	
9	answer			return		
10	shops	1	1	nurse	1	1
11	sight		1	spring		
12	joke			mind		1
	**TOTAL (TR)**				(T1) **7**	(T2) **8**
	**TOTAL (UN)**				(T1) **7**	(T2) **8**

Table S2: Example allocation of trained and untrained items. Each row is a word pair. A '1' in columns T1 and T2 denotes correct production of the word. Green: words allocated to the trained items list; red: words allocated to the untrained items list.

4. Spoken Picture Description tests

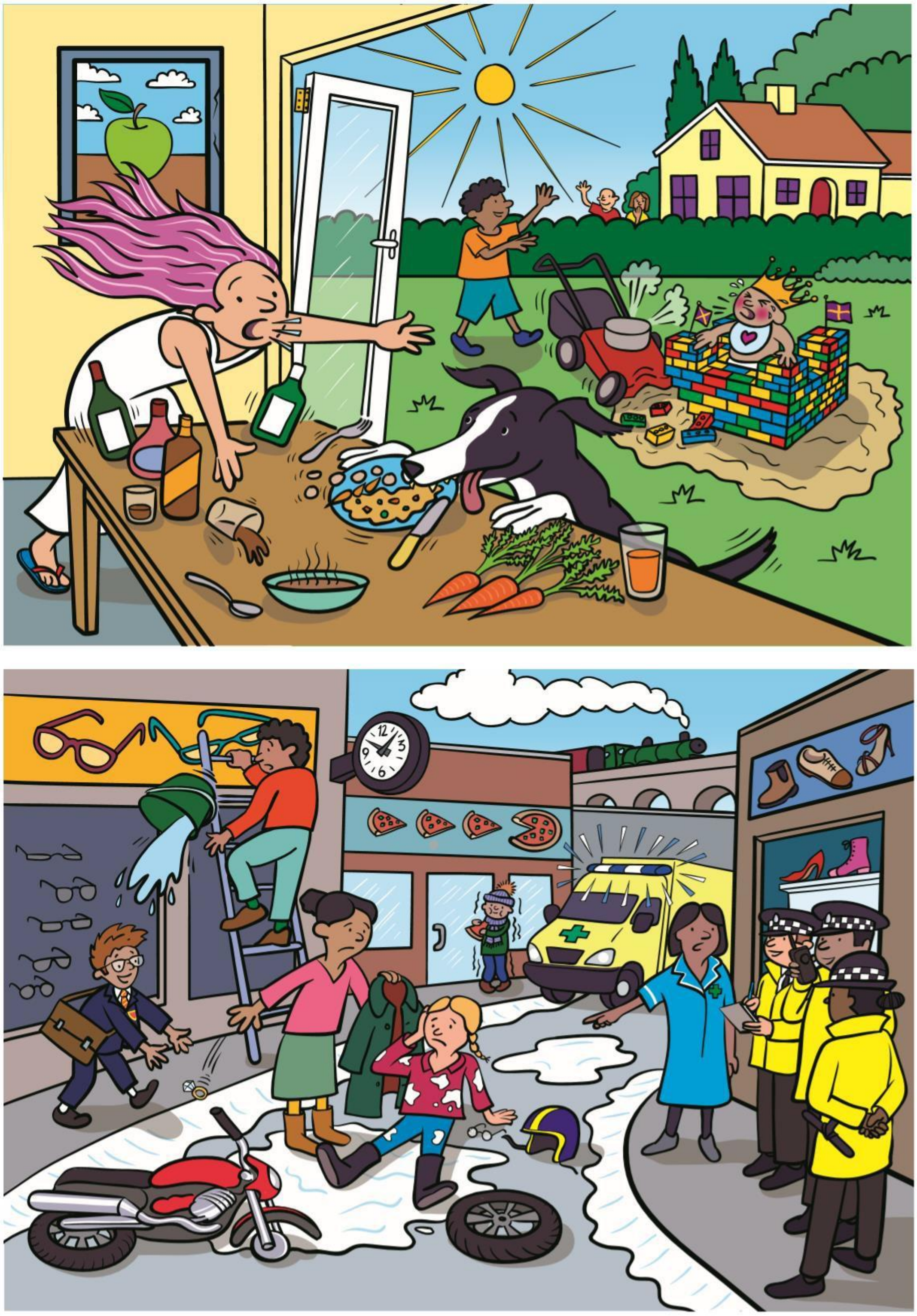

Figure S3: Spoken Picture Description. Top: Scene 1. Bottom: Scene 2

5. Sample size calculation (taken from the study protocol)

iTalkbetter: Average, one sample calculation, (comparison will be within group) based at three month follow up data (change in total aphasia severity score (the WAB) (taken from Katz, 1997)[6]). Change in WAB after therapy block compared with no therapy block = 1.5 [SD:3.3]; Alpha error= 5%; Power = 80%. Sample Size = 30. Expect 15% drop out so target = 35 patients in total.

We got close to our target of 30 patients completed as our drop-out rate was a little lower than expected (10%). Covid-19 pauses and end-of-funding considerations fed into our decision to stop recruitment at 32 participants.

6. Protocol

A 47 page protocol document is available from the PI (Prof Alex Leff) and will be made available on request.

7. References

1. Swinburn K, Porter G, Howard D. Comprehensive Aphasia Test: Psychology Press; 2004.
2. Conroy P, Sage K, Ralph MAL. The effects of decreasing and increasing cue therapy on improving naming speed and accuracy for verbs and nouns in aphasia. *Aphasiology* 2009; **23**(6): 707-30.
3. Nardo D, Holland R, Leff AP, Price CJ, Crinion JT. Less is more: neural mechanisms underlying anomia treatment in chronic aphasic patients. *Brain* 2017; **140**(11): 3039-54.
4. Barbera DS, Huckvale M, Fleming V, et al. NUVA: A Naming Utterance Verifier for Aphasia Treatment. *Comput Speech Lang* 2021; **69**.
5. Mckissock S, Ward J. Do errors matter? Errorless and errorful learning in anomic picture naming. *Neuropsychological Rehabilitation* 2007; **17**(3): 355 - 73.
6. Katz RC, Wertz RT. The efficacy of computer-provided reading treatment for chronic aphasic adults. *J Speech Lang Hear Res* 1997; **40**(3): 493-507.
