# Supplementary material for: Efficacy of a gamified digital therapy for speech production in people with chronic aphasia (iTalkBetter): behavioural and imaging outcomes of a phase II item-randomised clinical trial
Source: eClinicalMedicine. 2024 Feb 21;70:102483. doi: 10.1016/j.eclinm.2024.102483 (PMC11056404; doi:10.1016/j.eclinm.2024.102483)
Supplement: CIP_Phase 2_V2 [file mmc2.doc]

### Clinical Investigational Plan

**DINR**

| **Full title of Investigation:** | Digital Interventions in Neuro-Rehabilitation: Two digital neuro interventions (DNIs) for word retrieval. The development and testing of two web-based therapy applications for people with naming difficulties caused by Stroke (iTALKbetter) or mild- moderate Dementia (Gotcha!) in a small scale randomised trial. |
| --- | --- |
| **Short title:** | Digital Interventions in Neuro-Rehabilitation (DINR).   1. iTALKbetter |
| **Version and date of Clinical Investigation Plan (CIP):** | Version 2.0, 31.07.18 |
| **Sponsor:** | University College London (UCL) |
| **Sponsor CIP number:** | 18/0071 |
| **Funder (s):** | NIHR |

**Revision History:**

| **Version numbers** | **Date** | Summary of revisions in the case of amendments | Protocol Updated by [insert name & Signature] |
| --- | --- | --- | --- |
| 1 | 18.03.2018 |  |  |
| **2** | **31.07.2018** |  | Henry Coley-Fisher  Dr. Catherine Doogan |
|  |  |  |  |
|  |  |  |  |

# Signatures

The Chief Investigator (CI) and the JRO have discussed this Clinical Investigation Plan (CIP). The investigator agrees to perform the investigations and to abide by this CIP.

The investigator agrees to conduct the Investigation in compliance with the approved CIP, EU Good Clinical Practice (GCP) and UK Regulations for Devices (SI 2002/618; as amended) for regulated studies, the UK Data Protection Act (1998), the Trust Information Governance Policy (or other local equivalent), the Research Governance Framework (2005 2^nd^ Edition; as amended), the Sponsor’s SOPs, and other regulatory requirements as amended.

| **Chief investigator**  Prof Alex Leff | 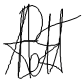 | 31.07.2018 |
| --- | --- | --- |
|  | Signature | Date |
| **Sponsor Representative** |  |  |
| UCL | Signature | Date |
|  |  |  |

Contents

[Clinical Investigational Plan 1](#__RefHeading___Toc516053445)

[Signatures 3](#__RefHeading___Toc516053446)

[Sponsor 9](#__RefHeading___Toc516053447)

[Principal Investigator, Coordinating Investigator and Investigation site(s) 9](#__RefHeading___Toc516053448)

[A.1 Overall Synopsis of Clinical Investigation 10](#__RefHeading___Toc516053449)

[*Inclusion criteria* 12](#__RefHeading___Toc516053450)

[Phase 2 12](#__RefHeading___Toc516053451)

[Phase 3 12](#__RefHeading___Toc516053452)

[*b) Exclusion criteria Phase 2 & 3* 13](#__RefHeading___Toc516053453)

[No diagnosis of developmental language disorders 13](#__RefHeading___Toc516053454)

[A.2 Background and Rationale 14](#__RefHeading___Toc516053455)

[A.3 Identification and description of the Investigational Device 15](#__RefHeading___Toc516053456)

[A.4 Justification for the design of the clinical investigation 17](#__RefHeading___Toc516053457)

[What new information will the research provide? 18](#__RefHeading___Toc516053458)

[A.5 Risks and benefits of the Investigational device and clinical Investigation 19](#__RefHeading___Toc516053459)

[A.6 Objectives and hypotheses of the clinical investigation 21](#__RefHeading___Toc516053460)

[A6.1 Hypotheses 21](#__RefHeading___Toc516053461)

[A6.2 Primary Objective 21](#__RefHeading___Toc516053462)

[Phase 2 21](#__RefHeading___Toc516053463)

[Phase 3 21](#__RefHeading___Toc516053464)

[A6.3 Secondary Objective(s) 21](#__RefHeading___Toc516053465)

[Phase 2 21](#__RefHeading___Toc516053466)

[iTALKbetter (only) 22](#__RefHeading___Toc516053467)

[• Which version of iTALKbetter optimises recovery of naming accuracy? 22](#__RefHeading___Toc516053468)

[Gotcha! (only) 22](#__RefHeading___Toc516053469)

[Phase 3 22](#__RefHeading___Toc516053470)

[A.7 Design of the clinical investigation 22](#__RefHeading___Toc516053471)

[A.7.1 General 22](#__RefHeading___Toc516053472)

[Phase 2: RCT (iTALKbetter) 22](#__RefHeading___Toc516053473)

[The on-line language battery (Comprehensive Aphasia Test) will be be audio and video recorded to ensure accuracy when scoring. Other tests of language will also be recorded. 23](#__RefHeading___Toc516053474)

[Phase 2: RCT Gotcha! 23](#__RefHeading___Toc516053475)

[Phase 3 (iTALKbetter & Gotcha!) 24](#__RefHeading___Toc516053476)

[A.7.2 Investigational device and comparators 24](#__RefHeading___Toc516053477)

[A.7.3 Subjects 24](#__RefHeading___Toc516053478)

[*a) Inclusion criteria* 24](#__RefHeading___Toc516053479)

[Phase 2 24](#__RefHeading___Toc516053480)

[Phase 3 24](#__RefHeading___Toc516053481)

[*b) Exclusion criteria Phase 2 & 3* 25](#__RefHeading___Toc516053482)

[No diagnosis of developmental language disorders 25](#__RefHeading___Toc516053483)

[*c) Criteria and procedures for subject withdrawal or discontinuation* 25](#__RefHeading___Toc516053484)

[*d) Point of enrolment.* 25](#__RefHeading___Toc516053485)

[*e) Total expected duration of the clinical investigation* 25](#__RefHeading___Toc516053486)

[*f) Expected duration of each subject's participation* 25](#__RefHeading___Toc516053487)

[*g) Number of subjects included in the clinical investigation* 25](#__RefHeading___Toc516053488)

[*h) Estimated time needed to select this number (i.e. enrolment period)* 25](#__RefHeading___Toc516053489)

[**Subject Eligibility** 25](#__RefHeading___Toc516053490)

[A7.4 Recruitment 26](#__RefHeading___Toc516053491)

[A7.5 Randomisation Procedures 27](#__RefHeading___Toc516053492)

[A.7.6 Procedures 27](#__RefHeading___Toc516053493)

[A.8 Informed Consent Process 28](#__RefHeading___Toc516053494)

[Informed consent and aphasia 28](#__RefHeading___Toc516053495)

[Procedure of gaining consent 29](#__RefHeading___Toc516053496)

[A.9 Schedule of assessments and interventions by visit 30](#__RefHeading___Toc516053497)

[A9.1 Laboratory Assessments and Procedures 31](#__RefHeading___Toc516053498)

[A.10 Device accountability 31](#__RefHeading___Toc516053499)

[A.11 Monitoring Plan 31](#__RefHeading___Toc516053500)

[a. Confidentiality 32](#__RefHeading___Toc516053501)

[b. Record keeping and archiving 32](#__RefHeading___Toc516053502)

[A.12 Statistical Considerations 32](#__RefHeading___Toc516053503)

[**Design** 32](#__RefHeading___Toc516053504)

[A small, well-defined sample of patients with the potential to benefit from the DNI’s will be recruited. In both cases the main outcome measure is whether the DNI is effective at improving naming impairments. This comparison is within-subject and is achieved by comparing post-therapy measures to multiple baseline measures. A secondary question is whether the therapy can be optimized so in each case we are comparing two different versions of the therapy (a between group comparison). So, in both trials patients are randomized into one of the two types of therapy. 32](#__RefHeading___Toc516053505)

[A.13 Data Management 34](#__RefHeading___Toc516053506)

[A13.1 Procedures for data review, database cleaning, and issuing and resolving data queries. 35](#__RefHeading___Toc516053507)

[A13.2 Procedures for verification, validation and securing of electronic clinical data systems 35](#__RefHeading___Toc516053508)

[A13.3 Data retention 36](#__RefHeading___Toc516053509)

[A13.4 Clinical quality assurance 36](#__RefHeading___Toc516053510)

[A13.5 Completion of Case Report Forms 36](#__RefHeading___Toc516053511)

[A13.6 Review and Return of Completed Documentation 36](#__RefHeading___Toc516053512)

[A13.7 Retention of Documentation 36](#__RefHeading___Toc516053513)

[A13.8 Training 37](#__RefHeading___Toc516053514)

[A.14 Amendments to the CIP 37](#__RefHeading___Toc516053515)

[A.15 Deviations from clinical investigation plan 38](#__RefHeading___Toc516053516)

[A.15.1 Procedures for recording, reporting and analysing CIP deviations 38](#__RefHeading___Toc516053517)

[A.15.2 Procedure for reporting any protocol deviations 38](#__RefHeading___Toc516053518)

[The clinical investigation shall not commence recruitment until all REC, regulatory (if applicable) and local (NHS permission) is received. All additional requirements imposed by the REC or regulatory authority will be followed. 39](#__RefHeading___Toc516053519)

[A.16 Insurance 39](#__RefHeading___Toc516053520)

[A.17 Adverse events, adverse device effects and device deficiencies 39](#__RefHeading___Toc516053521)

[a-c) Definitions 39](#__RefHeading___Toc516053522)

[An adverse event does not include: 40](#__RefHeading___Toc516053523)

[d) Reporting requirements and timelines 40](#__RefHeading___Toc516053524)

[e) Assessments of adverse events 42](#__RefHeading___Toc516053525)

[Seriousness 42](#__RefHeading___Toc516053526)

[Causality 42](#__RefHeading___Toc516053527)

[Expectedness 43](#__RefHeading___Toc516053528)

[f) Procedures for recording and reporting Adverse Events and Device Deficiencies 43](#__RefHeading___Toc516053529)

[Investigator responsibilities: 43](#__RefHeading___Toc516053530)

[Reporting of all Adverse Events and Device Deficiencies: Investigator and Sponsor responsibilities 44](#__RefHeading___Toc516053531)

[Progress reports 44](#__RefHeading___Toc516053532)

[a) Anticipated adverse device effects, together with their likely incidence, mitigation or treatment. 44](#__RefHeading___Toc516053533)

[b) Outline Information regarding an IDMC, if established. This is not yet established. 45](#__RefHeading___Toc516053534)

[A.18 Oversight Committees 45](#__RefHeading___Toc516053535)

[A.19 Vulnerable population 45](#__RefHeading___Toc516053536)

[A.20 Suspension or premature termination of the clinical investigation 46](#__RefHeading___Toc516053537)

[a) Criteria and arrangements for suspension or premature termination of the whole clinical investigation or of the clinical investigation in one or more investigation sites. 46](#__RefHeading___Toc516053538)

[c) Requirements for subject follow-up. 46](#__RefHeading___Toc516053539)

[A20.1 Subject Withdrawals and Discontinuation 47](#__RefHeading___Toc516053540)

[A.21 Definition of End of Trial 47](#__RefHeading___Toc516053541)

[A.22 Publication policy 47](#__RefHeading___Toc516053542)

[A.23 Bibliography 49](#__RefHeading___Toc516053543)

**List of abbreviations**

| ADE | Adverse Device Effect |
| --- | --- |
| AE | Adverse Event |
| CA | Competent Authority |
| CI | Chief Investigator |
| CIA | Clinical Investigation Agreement |
| CIP | Clinical Investigation Plan |
| CRF | Case Report Form |
| CRO | Contract Research Organisation |
| DINR | Digital interventions in Neuro-rehabilitation |
| DNI | Digital Neuro-intervention |
| DCF | Data Clarification Form |
| DD | Device Deficiency |
| EC | European Commission |
| EU | European Union |
| EUDAMED | European Medical Devices Regulatory Database |
| G! | Gotcha! |
| G!m | Gotcha! Maintenance |
| GCP | Good Clinical Practice |
| GMP | Good Manufacturing Practice |
| HRA | Health Research Authority |
| IB | Investigator Brochure |
| ICF | Informed Consent Form |
| IMD | Investigational Medical Device |
| ISF | Investigator Site File |
| JRO | Joint Research Office |
| MA | Marketing Authorisation |
| MHRA | Medicines and Healthcare products Regulatory Agency |
| NCA | National Competent Authority |
| NHS | National Health Service |
| NHS R&D | National Health Service Research & Development |
| NICE | National Institute of Clinical Excellence |
| NIHR | National Institute of Health Research |
| NIST | National Institute of Standards and Technology |
| PI | Principal Investigator |
| PIS | Participant Information Sheet |
| QA | Quality Assurance |
| QC | Quality Control |
| SADE | Serious Adverse Device Effect |
| SAE | Serious Adverse Event |
| SALT | Speech and Language Therapy |
| SOP | Standard Operating Procedure |
| TMG | Trial Management Group |
| UADE | Unanticipated Adverse Device Effect |
| UK | United Kingdom |
| USADE | Unanticipated Serious Adverse Device Effect |
| WTCN | Wellcome Trust Centre for Neuroimaging |

# Sponsor

| Sponsor’s representative  Shriram Velamuri | Joint Research Office, UCL, 1st Floor Maple House,  149 Tottenham Court Road,  London W1T 7NF  Postal address:  Joint Research Office, UCL  Gower Street,  London WC1E 6BT |
| --- | --- |

# Principal Investigator, Coordinating Investigator and Investigation site(s)

| Chief Investigator (CI)  Professor of Neurology | Prof Alex Leff,17-19 Alexandra House, Queen Square, WC1N 3AZ  [a.leff@ucl.ac.uk](mailto:a.leff@ucl.ac.uk)  0207 279 1129 |
| --- | --- |
| Principal Investigator (PI)  Professor of Cognitive Neurology  & Honorary Consultant Neurologist | Prof Alex Leff,17-19 Alexandra House, Queen Square, WC1N 3AZ  [a.leff@ucl.ac.uk](mailto:a.leff@ucl.ac.uk)  0207 279 1129 |
| Co-Investigator | Dr. Catherine Doogan, UCL, [c.doogan@ucl.ac.uk](mailto:c.doogan@ucl.ac.uk), (Clinical Psychologist)  Dr. Jennifer Crinion, UCL, [j.crinion@ucl.ac.uk](mailto:j.crinion@ucl.ac.uk) (Speech and Language therapist)  Henry Coley-Fisher, [henry.coley-fisher.15@ucl.ac.uk](mailto:henry.coley-fisher.15@ucl.ac.uk), Research Assistant,  NeurotherapeuticsLab  ICN 17-19 Queen Square, London, WC1N 3AZ  0207 279 1134 |
| Add in other involved investigators as required | Wellcome Centre for Human Neuroimaging (WCHN), Institute of Neurology, UCL  Head of Center Prof Cathy Price. |
| Statistician | Dr Tom Hope,  Senior Research Associate  Wellcome Centre for Human Neuroimaging  University College London  London WC1N 3AR |

# A.1 Overall Synopsis of Clinical Investigation

| **Title:** | Digital Interventions in Neuro-Rehabilitation (DINR): Two  digital neuro interventions (DNIs) for word retrieval. The development and testing of two web-based therapy applications for people with naming difficulties caused by Stroke (iTALKbetter) or mild- moderate Dementia (Gotcha!). |
| --- | --- |
| **Short title:** | Digital Interventions in Neuro-Rehabilitation (DINR): iTALKbetter & Gotcha! |
| **Device:** | Two digital interventions in Neuro-rehab (DNIs). One word-retrieval app for people with Stroke and one word-retrieval app for people with mild-moderate Alzheimer’s disease. |
| **Objectives:** | Digital Interventions in Neuro-Rehabilitation (DINR): Two digital neuro interventions (DNIs) for word retrieval. The development and testing of two web-based therapy applications for people with naming difficulties through a small scale randomized controlled trial.  **iTALKBetter** will provide an app-based therapy for people with word retrieval difficulties caused by stroke (naming app for a wide variety of common words and phrases).  For both DNIs the main outcome measure will be a clinically relevant improvement on the naming ability of the trained items (compared to untrained control items).  Secondary outcomes will include improvements in social activity and participation and participant and/or carer reported outcome measures (PROMS).  For both DNIs we also plan to capture brain-based data (functional and structural MRI and MEG) in order to test a series of secondary hypotheses relating to both how and why the therapy may work in some participants but not in others. |
| **Type of Investigation:** | Two Class I randomized control trials both single-site.  Investigation in stroke and Dementia. |
| **Investigation design and methods:** | This is a summary of the protocol for the study funded by the National Institute of Health Research (NIHR). This project has 3 phases. Phase 1 has been granted ethical approval by the Queen square REC (reference: 17/LO/1846). This application seeks ethical approval for **phases 2 only**. The main aim of phase 1 (17/LO/1846) was to develop two novel, web-based, digital neuro-interventions (DNIs) funded by the NIHR. The main aim of phase 2 is to test the clinical efficacy of these DNIs through a trial. When the trial is finished we will analyse the data we will then seek CE marking if it is effective. The aim of phase 3 is to ‘roll-out’ the DNIs making them available online. The participants will take the app home on a mobile device and ‘play’ the therapy. After the trial the participant will return the device and we will delete any content on the device and reload the DNI.  **Phase 2** of these studies are randomised small-scale trials.  **iTALKbetter**: iTALKbetter is a small-scale, randomised, clinical trial for participants with post-stroke aphasia, namely impaired word retrieval for things. Participants will be randomized (using minimization) to one of two versions of the therapy: 1) deterministic iTALKbetter, 2) reactive iTALKbetter.  Participants will complete 5 assessment sessions (T1-T5) at UCL – they will complete neuropsychological assessments receive brain imaging scans, and complete a program of therapy using the DNI.  While both DNIs (apps) will be aimed at different participant populations, the underlying software and development pipelines are almost identical, hence our combining them here for the purposes of ethical review; both apps treat word retrieval. We make it clear at all points where there are any points of difference. |
| **Investigation duration per participant:** | iTALKBetter: **30 weeks** |
| **Estimated total Investigation duration:** | iTALKBetter: **3 years** |
| **Planned Investigation sites:** | Single-site is **UCL**. |
| **Total number of participants planned:** | iTALKBetter: 35 |
| **Main inclusion/exclusion criteria:** | *Inclusion criteria*Phase 2 Any type of stroke but at least 6 months after onset or person with mild/moderate dementia  Evidence of aphasia on the Comprehensive Aphasia Test (Swinburn, 2004) or self-reported proper naming difficulty which we will then assess at Time Point 1  English as their dominant language  Able to tolerate MRI brain scan  Able to give informed consent  Able to use the DNI (app) Phase 3 Any type of stroke  Evidence of aphasia or proper naming difficulty on the baseline tests.  English as their dominant language *b) Exclusion criteria Phase 2 & 3*No diagnosis of developmental language disorders No diagnosis of severe dementia or primary progressive aphasia  No major co-existing neurological or psychiatric diagnosis  No contraindications to brain scanner (Phase 2 only e.g. the presence of ferromagnetic implants or other metallic or electronic objects in the body,  weight over 24 stone, claustrophobia or pregnancy). |
| **Statistical methodology and analysis:** | **Behavioural data:**  Both DNIs: Behavioural baseline data will be analysed using to ensure that the two groups are not unbalanced on key demographic variables, including baseline performance during the randomization procedure. This process of randomization is called minimization (Altman, BMJ 2005).  iTALKbetter: Data from all timepoints will be analysed using a repeated-measures ANOVA to determine whether there is an interaction between time (therapy block vs baseline) and item (trained vs untrained). The effect of the between-group factor (therapy type) is a secondary outcome measure which will also be analysed using a repeated measure ANOVA but with this extra factor added in: more formally, a three-way interaction between time (therapy block vs baseline), item (trained vs untrained) and therapy type (iTB: deterministic vs reactive).  **Neuroimaging data:**  Collected at three of the time points. Structural magnetic resonance imaging (MRI) data will be used to identify the lesion location and to aid co-registration of the functional MEG data. Brain structure at the three time points will be analysed using Voxel-Based Morphometry (Ashburner and Friston 2000) within the Statistical Parametric Mapping software (SPM12) to identify whether these therapies induce any structure brain changes over time. We will employ multi- parameter mapping MRI methodology for the structural brain scans (Callaghan et al, 2014). |

# A.2 Background and Rationale

**Background**

**Stroke**

Mortality from stroke in the UK has reduced from 21% in 1999 to 12% in 2008. However, stroke prevalence has been increasing, the consequence of socioeconomic and scientific advances that have improved survival; which means that more people are surviving with long-standing disability. Language impairment (aphasia) is the second most common major impairment after stroke, with a prevalence of 250,000 in the UK. Aphasia may respond to therapy many months and years after the stroke occurs, but provision of specialist therapy (speech and language therapy - SALT) is far below that needed to provide optimal rehabilitation (Code and Heron, 2003).

We will address this by targeting a common symptom of post-stroke aphasia: impaired word retrieval problems. This is particularly important in patients receiving rehabilitation for associated disabilities as poor speech production can impair participation/compliance with treatment programmes. Our study is designed to improve word retrieval in patients with post-stroke aphasia, who are in the chronic phase (>6 months post-stroke).

The main aim is to develop and test the clinical efficacy of a novel, web-based, DNI. iTALKbetter will provide an effective training tool that patients can use to practice independently. This will free-up SALT time to provide additional assessment, supervision and functional intervention in a highly cost effective manner.

1. **iTALKbetter** will provide an app-based therapy for people with word retrieval difficulties caused by stroke (naming app for a wide variety of common words and phrases).

These two Digital Neuro Interventions (DNIs) will provide the opportunity for the necessary increased rehabilitation that help people recover lost function due to brain disease. This will alleviate NHS Speech and Language Therapist (SALT) time and put users in control of when and where they carry out practice-based language therapy.

Only clinical trial of a similar digital therapy is of Step-by-Step (aphasia software that is CE marked). a small-scale study of this (which had positive therapeutic effects) was published in stroke: The CACTUS trial:

Palmer, R., Enderby, P., Cooper, C., Latimer, N., Julious, S., Paterson, G., ... & Delaney, A. (2012). Computer therapy compared with usual care for people with long-standing aphasia poststroke: a pilot randomized controlled trial. *Stroke*, *43*(7), 1904-1911.

# A.3 Identification and description of the Investigational Device

Here is a summary of our two naming eTherapies, also called Digital Neuro Interventions.

**Purpose**

Both eTherapies are to help patients with word retrieval problems. iTALKbetter is for patients with aphasia, usually caused by a stroke; Gotcha! is for patients with mild to moderate dementia who have trouble naming familiar people.

**What do they do?**

Both eTherapies work via mass practice and feedback to the users on a trial-by-trial basis. The eTherapy is very simple in a way; it just presents a long series of pictures for users to name. Both eTherapies will use voice recognition (VR) software in order to make a binary decision as to whether the user said the correct word or not. This affects what the next trial (object to name) is and what auditory cue (if any) is provided the next time the user has to name the same item (see below):


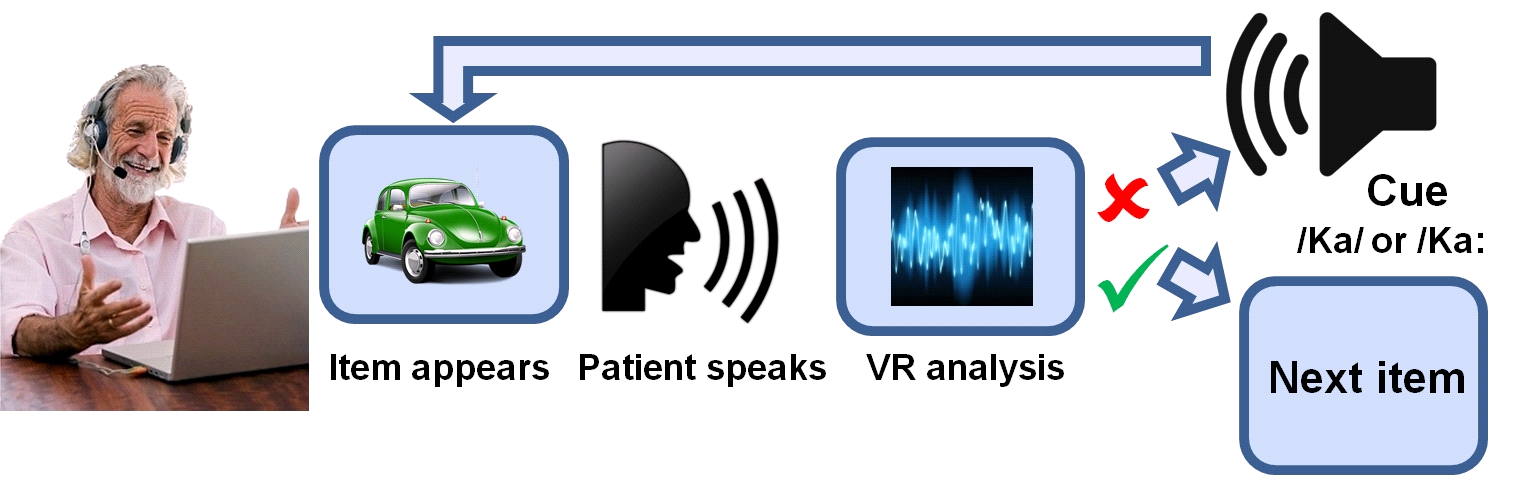


*The speech will be recorded on the device but not identifiable information will be stored on it but when the participant finished the trial we will delete all information on the device.

**What are the scientific phases of development?**

**Phase 0:** A prototype of the therapy is developed by the research and software team. This has the key elements of the therapy built in (pictures to-be-named, voice recognition software, auditory cues, how pictures are recycled). We have a current version for iTALKbetter but not for Gotcha! (although Gotcha! has almost identical mechanics to it).

**Phase 1:** The aim is to improve the prototype, primarily its usability and accessibility so suitable patients will use in the phase 2 trail. This is an iterative process between the three key stakeholders: a) the patients and their relatives; b) the scientific team; c) the software team. This software team SoftV has been used by Prof Leff previously and have a contract with him through UCL. At the end of this phase we have a version of the therapy to go into the trial phase.

**Phase 2:** Exploratory trial. A small, well-defined sample of patients with the potential to benefit from the eTherapies will be recruited. In both cases the main outcome measure is whether the DNI is effective at improving naming impairments. This comparison is within-subject and is achieved by comparing post-therapy measures to multiple baseline measures. A secondary question is whether the therapy can be optimized so in each case we are comparing two different versions of the therapy (a between group comparison). So, in both trials patients are randomized into one of the two types of therapy. Outcome measures are collected by the research team using a variety of standardized and non-standardized tests of language and cognition. These outcome measures are not built into the eTherapy at this point.

**Phase 3:** Roll out. Informed by the phase 2 trial, the eTherapy is updated to make it more efficient (e.g.: picture recycling, how often new pictures are bought into the therapy, etc.). At this point, outcome measures are now added into the eTherapies so that they become ‘stand alone’. This means that individual users can both practice with the therapy, and see if it is having an effect for them. We also collect and analyse the data (in a secure, pseudoanonymized database) for scientific publication and further refinement of the therapy algorithms. Participants in this phase are self-selecting, and could come from any part of the world (assuming they have internet access and hardware of their own to run the eTherapy).

The interventions which will be an application (DNI) on a tablet (iTALKbetter) will be introduced to the participant when attending a testing session. One of the research team will explain how to use the DNI and give some time for practice items. When confident that the participant can use it independently then the participant may take this device home and continue their therapy (suggested therapy time is 5-10 hours per week) which will be simultaneously monitored by the research team. However, the research team will check in on them weekly to trouble shoot any difficulties they may have whether motivational or technical. When the participant has completed the therapy block then we will invite them in again for testing and they will return the mobile device with the DNI on it. The participants who continue to receive standard care during the therapy block will be asked to record and submit details to the research team. After Phase 2 has been completed the participants will be able to access the DNI for free.

Any traceablity issue we will have is to collect which version of the therapy they have received in Phase 2.

# A.4 Justification for the design of the clinical investigation

**Why is the research considered worth doing?**

Standard (face to face) Speech and Language Therapy (SALT) has a huge evidence base but patients in the NHS have unmet therapy needs due to a lack of resources. The evidence from speech-therapy highlights the amount of time-on-task required to improve patients' ability to communicate. Naming problems are common and impact on the patients’ wellbeing and social inclusion. There is also good evidence that current existing therapeutic approaches work.

A Cochrane review of 39 RCT's involving 2518 participants concluded that speech-therapy results in significant benefits to patients' functional communication (Brady, 2012). Many of these studies involve high doses of therapy. A meta-analysis examining dose found that positive therapy studies averaged 98 hours of speech therapy in total, while negative studies averaged 43 (Bhogal, 2003). In the NHS a patient with aphasia can expect a total average of 6-10 hours of SALT (Code, 2003). When the intervention takes place or how intensely seems less relevant to recovery (Moss, 2006). Our solution to the lack of available SALT therapy is to produce digital neuro-interventions that give patients the opportunity to practice scientifically validated, impairment based therapy when and where it suits them which gives them access to optimum therapy dose.

Recent improvements in acute stroke care and the ever-growing population of people with dementia means that demand for already limited NHS resources will increase. Commissioners will need to address this growing unmet need and innovative relatively cheap interventions like this one will provide necessary rehabilitation.

We know that therapy is effortful for patients who can sometimes be too fatigued when the therapist comes to do their session. These digital neuro-interventions will provide optimum availability of therapy and therefore place the patient in control of how much they do. However, as we recognise it is still effortful we hope through to make the therapy engaging through adaptive and exciting software. The adaptiveness will serve to reward the patients for their efforts without ever making them feel like they are failing or doing unwell. This will hopefully boost how long they spend in therapy and therefore make greater gains.

There are therapy 'apps" available for patients with aphasia but the scientific basis for them is lacking and none are targeted for speech production. By using voice recognition software our interventions will be uniquely designed to be used by patients with aphasia.

### What new information will the research provide?

The concept of the therapy component is based on standard SALT practice and thus has a strong proof of concept basis, as do our proposed outcome measures. The novel component is packaging it in a user- friendly web-app that is designed for and by people with word-retrieval problems. We will test the efficacy in a randomized-controlled trial (Phase 2) and then in a pragmatic real world trial (Phase 3). This is ambitious and yet we believe a realistic goal to provide a world-wide evidence based therapy that can benefit many. These digital neuro-interventions will be in contrast to the plethora of apps available on line claiming cognitive training without having scientific proof. During Phase 3 we will continue to collect and analyse data to determine the clinical effectiveness of the interventions when they are rolled out and made available to all. This roll-out phase is rare and therefore will provide significant unique information that cannot be found in small scale trials.

Phase 2 (London) for iTALKbetter is a small-scale, randomised, clinical trial for participants with post-stroke aphasia, namely impaired word retrieval for things. The main research question is whether iTALKbetter improves patients’ naming ability for trained items. We test this hypothesis by assessing participants naming abilities on two matched lists of items, half are trained items and half are untrained. A secondary question is whether it matters how the software progresses patients through the therapy. We will test this hypothesis by creating two different versions of iTALKbetter: ‘deterministic’ and ‘reactive’. Participants will be randomized (using minimisastion) to one of two of these versions of the therapy. Participants practice with the therapy for set periods of time (therapy block) following a series of (three) baseline measures split over a single, pre-therapy block (see Trial design section 5). The main outcome measure will be a clinically relevant improvement on the naming ability of the trained items (compared to untrained control items). Secondary outcomes include investigating which therapy version optimises recovery on naming accuracy and also improvements in social activity and participation and participant and/or carer reported outcome measures (PROMS).

For both DNIs we also plan to capture brain-based data (functional and structural MRI and MEG) in order to test a series of secondary hypotheses relating to both how and why the therapy may work in some participants but not others. All brain imaging will be outside of standard care for the participants and take place at UCL by trained staff.

Phase 3 Will be the roll-out of the DNI therapy applications on the internet with a pragmatic trial of whether therapy gains can be made outside the confines of a phase II clinical trial. The comparison will be on similar outcome measures as phase II with a control test on sustained attention (internal control) that we predict will not improve with therapy. As part of this phase we will improve the therapy by testing two different versions of the therapy by randomly allocating incoming users to version A or B. This methodology is commonly used in the gaming industry and in psychological experimentation (where it is known as split testing).

# A.5 Risks and benefits of the Investigational device and clinical Investigation

The potential clinical benefit to the participants is that they will be receiving more therapy through the DNI that they could ever receive from the NHS. This in turn will be provide opportunity for them to improve their word retrieval which can be distressing impairment following stroke and the onset of Alzheimer’s dementia.

The table below summaries the risks and mitigations of **the investigational procedures** that are being performed:

**Table 1**

| Name of IMD | Potential risk | Risk Frequency | Risk Management |
| --- | --- | --- | --- |
| DNIs | Each DNI will be delivered via a tablet or mobile device. Potential risks from prolonged screen watching:  Headaches, eyestrain, fatigue | Daily during trial | Participants will be encouraged to take regular beaks and to avoid prolonged screen time. |
| DNIs | Increased awareness of the extent of the impairment through testing and playing DNI. | Daily during trial (because participants get trial-by-trial feedback on performance) | Participants will be encouraged to talk to clinical psychologist (co- investigator). |

The table below summaries the risks and mitigations of all test above standard care that are being performed:

**Table 2**

| Intervention | Potential risk | Risk Management |
| --- | --- | --- |
| MRI | Only if any contraindications are missed or not reported.  These contraindications may include the presence of any metal in the body.  Discomfort or anxiety in the MRI scanner may occur due to the noise and enclosed space. | Performed by trained member of staff.  Follow trust standard operational procedures by obtaining surgical history of exposure to metal, metallic dental work and other potential MRI contraindications.  These potential risks will be reduced by providing an alarm bulb in the case of any uncertainty discomfort, or distress. Noise from the scanner will be attenuated by the use of earplugs and noise- shielding headphones |
| X-Ray | Exposure to ionising radiation | Performed by trained member of staff. Following trust protocols.  The standard dose is equivalent to ~0.005% of average annual radiation dose in the UK |

The classification of medical devices in the European Union is outlined in Annex IX of the Council Directive 93/42/EEC (as amended). There are four classes, ranging from low risk to high risk.

Class I

Class IIa

Class IIb

Class III

The Medical Device used in this investigation is classified as **Class I**

# A.6 Objectives and hypotheses of the clinical investigation

## *A6.1 Hypotheses*

## *A6.2 Primary Objective*

All objectives are similar for both iTALKbetter and Gotcha!

**Primary Objectives**

### Phase 2

Do these DNIs provide a clinically meaningful improvement in word-retrieval and language function in people with stroke and dementia, in a small-scale, randomized controlled trial?

### Phase 3

When accessible as an online app, does practice with each DNI provide clinically meaningful improvements in word retrieval and language function in people with stroke and dementia?

## *A6.3 Secondary Objective(s)*

We believe that using these DNI’s will improve their word retrieval on trained items by a known therapy used by SLT’s through a digital app and increased dose of therapy. However we also believe that by using this DNI and practicing every day that it will effect changes in their structural brain imaging and the health of the participants and their carers.

### Phase 2

- Are the therapy effects item-specific or do we see generalization to untrained items?
- Are the effects of DNI therapy limited to the language domain alone or do they improve other cognitive domains e.g. sustained attention?
- Do the DNIs improve participant’s social activity and participation?
- Are the DNIs acceptable to participants (can they use them easily)?
- Are the DNIs acceptable to participants’ carers?
- Do the DNIs improve carer health/well-being?
- Do the DNIs have any economic benefits?
- Can baseline structural brain imaging explain participants’ responses to each DNI?
- Can baseline structural brain imaging be used to predict incoming participants’ response to each DNI?
- Can repeated-measures structural or functional brain imaging identify participant’s brain areas that change in response to each neuro-intervention?

### iTALKbetter (only)

### Which version of iTALKbetter optimises recovery of naming accuracy?

### Phase 3

- Can each DNI maintain its clinical effectiveness while being delivered remotely?
- Can the clinical effectiveness of both therapies be improved by randomising participants to mechanistically different versions of the therapy?
- Do the DNIs improve participant’s social activity and participation?
- Do the DNIs have any economic benefits?

# A.7 Design of the clinical investigation

## *A.7.1 General*

### Phase 2: RCT (iTALKbetter)

iTALKbetter is a small-scale, randomised, clinical trial for participants with post-stroke aphasia, namely impaired word retrieval for things. Participants will be randomized (using minimisation) to one of two versions of the therapy: 1) deterministic iTALKbetter, 2) reactive iTALKbetter.

Participants will complete 5 assessment sessions (T1-T5) at UCL – they will complete neuropsychological assessments receive brain imaging scans, and complete a program of therapy using the DNI.


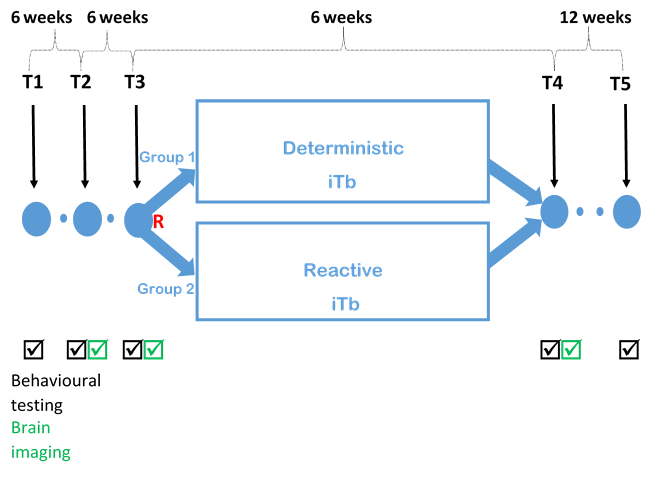


### The on-line language battery (Comprehensive Aphasia Test) will be be audio and video recorded to ensure accuracy when scoring. Other tests of language will also be recorded.

### Phase 3 (iTALKbetter & Gotcha!)

The post-trial internet version roll-out will be shaped by Phase 1 and 2.

In this phase self-selected patients will log on to the therapy website, sign an online consent form and then have access to electronic versions of the baseline and main language outcome tests as above. Once these are completed, they will be able to access the therapy for either naming things (iTALKbetter) or names of people (Gotcha!). After set amounts of therapy time, they will be prompted to test themselves again on the outcome measures. We will add in an outcome measure that we predict will not change with therapy (a test of sustained attention); this will be our control measure (to demonstrate specificity of the therapy in the online version). Participants will be able to access their own results but not of others. They can withdraw consent at any time.

Data will be stored on a secure UCL website and only members of the research team will be able to access the data to analyse it. This is the same set-up that Prof Leff has for two successful web- based therapies for patients with visual field disorders: Read-Right and Eye Search.

## *A.7.2 Investigational device and comparators*

The tablets and phones which the DNI will be uploaded onto will be stored in a secure cabinet in the ICN building. After the trial ends the DNI will be deleted from the app and the mobile devices will be stored or used by Prof Leff as he sees fit. No data will remain on the individual devices.

## *A.7.3 Subjects*

### *a) Inclusion criteria**

### Phase 2

Any type of stroke but at least 6 months after onset or person with mild/moderate dementia

Evidence of aphasia on the Comprehensive Aphasia Test (Swinburn, 2004) or self-reported proper naming difficulty which we will then assess at Time Point 1

English as their dominant language

Able to tolerate MRI brain scan

Able to give informed consent

### Phase 3

Any type of stroke or mild/moderate dementia

Evidence of aphasia or proper naming difficulty on the baseline tests.

English as their dominant language

### *b) Exclusion criteria Phase 2 & 3**

### No diagnosis of developmental language disorders

No diagnosis of severe dementia or primary progressive aphasia

No major co-existing neurological or psychiatric diagnosis

No contraindications to brain scanner (Phase 2 only e.g. the presence of ferromagnetic implants or other metallic or electronic objects in the body, weight over 24 stone, claustrophobia or pregnancy).

### *c) Criteria and procedures for subject withdrawal or discontinuation*

A participant may be withdrawn from the trial whenever continued participation is no longer in the participant’s best interests, but the reasons for doing so must be recorded. Reasons for discontinuing the trial may include:

- - - disease progression whilst in the study
    - intercurrent illness
    - patients withdrawing consent
    - persistent non-compliance to protocol requirements.

### *d) Point of enrolment.*

Participants are enrolled at T1 (first time point) as this is where consent is obtained. They are randomized at T3 (after all three baseline measurements have been collected).

### *e) Total expected duration of the clinical investigation*

This for both studies is 3 years.

### *f) Expected duration of each subject's participation*

iTALKbetter: 30 weeks

###

### *g) Number of subjects included in the clinical investigation*

iTALKbetter: 35

### *h) Estimated time needed to select this number (i.e. enrolment period)*

2.5 years

**Subject Eligibility**

Once written informed consent has been obtained, the Case Report Form will be completed to document adherence to the inclusion and exclusion criteria.

Where a subject fails to fulfil any element of the inclusion and exclusion criteria, this will be documented and the signed consent form and completed inclusion/exclusion criteria retained by the Principal Investigator. The subject will not be advanced any further into this clinical investigation.

**Subject Identification**

When a subject is identified and considered eligible for entry into this clinical investigation, the subject will be allocated the next available investigation number (subject ID number).

For subjects enrolled, this number will consist of 01 for the first subject, 02 for the second subject and so on. This number will be the unique identifier of the subject and written on each page of the paper/electronic Case Report Form booklet and all other documentation relating to that subject.

Each subject that is enrolled into the study will have their study participation recorded and details of the device recorded in their hospital notes, a copy of their signed consent form and patient information sheet should also be placed on his/her hospitals notes to identify the subject as participating in a Clinical Investigation.

## *A7.4 Recruitment*

Participant recruitment at a site will only commence when the trial has been initiated by the Sponsor (or it’s delegated representative), and issued with the ‘Open to Recruitment’ letter.

**iTALKbetter Phase 2**

Suitable participants will be identified by the clinical care team (Prof Leff, Dr. Crinion) and advertised in their clinics. If already consented to be contacted about participation in research then contacted by telephone by a member of the research team (usually Dr Doogan or a research assistant but may include other members of the team). Participants will be initially identified from UCL and UCLH clinics run by the clinicians on the clinical care team and existing databases that we have permission to use (e.g. the PLORAS stroke database in London CI: Professor Cathy Price, REC reference number 13/LO/1515). We may also advertise in local community rehab services and support groups. Participants who consent to be contacted by the research team will be consented to take part in the study at time-point 1.

**Phase 3** participants or users of the web-based versions will be self-recruiting.

## *A7.5 Randomisation Procedures*

**Phase 2 iTALKbetter**

Following participant consent, and confirmation of eligibility the randomisation procedure described below will be carried out.

After consent has been gained and the participants have completed 3 sessions of baseline testing then CI will randomise the participants using minimisation. This will determine which version of the therapies the participant will use: deterministic or reactive versions of the therapy. After time point 2 the participant will also be randomised to which list of words they are being trained on which will be computer generated.

Participants are considered to be enrolled into the trial following: consent, pre-treatment assessments, confirmation of eligibility, completion of the randomisation process, allocation of the participant trial number and treatment by the central coordinating team/remote system.

Participants are considered to be enrolled into the trial following: consent, pre-treatment assessments, confirmation of eligibility, allocation of the participant trial number and treatment by the central coordinating team/remote system. The completion of the randomisation process happens at time point 3 and so they will have been enrolled since Time point 1.

## *A.7.6 Procedures*

The interventions which will be an application (DNI) on a tablet (iTALKbetter) will be introduced to the participant when attending a testing session. One of the research team will explain how to use the DNI and give some time for practice items. When confident that the participant can use it independently then the participant may take this device home and continue their therapy (suggested therapy time is 5-10 hours per week) which will be simultaneously monitored by the research team. However, the research team will check in on them weekly to trouble shoot any difficulties they may have whether motivational or technical. When the participant has completed the therapy block then we will invite them in again for testing. The participants who continue to receive standard care during the therapy block will be asked to record and submit details to the research team.

Brain Imaging will also take place. For both DNIs this will happen 3 times, at time-point 1, 3 and 4. The scans will take place in UCL, carried out by trained staff, on the same day as the participants are coming in for behavioural testing and will last approximately 30 minutes.

# A.8 Informed Consent Process

We understand it is the responsibility of the Investigator, or a person delegated by the Investigator to obtain written informed consent from each subject prior to participation in the Investigation, following adequate explanation of the aims, methods, anticipated benefits and potential hazards of the study.

The person taking consent will be GCP trained, suitably qualified and experienced, and have been delegated this duty by the CI/PI on the delegation log.

A week will be given for consideration by the patient before taking part. The PI must record when the patient information sheet (PIS) has been given to the patient. [If the amount of time between the PIS being given and the date of consent are less than 24 hours, the PI will explain the rationale for this]. It must be recorded in the medical notes when the participant information sheet (PIS) has been given to the participant.

The Investigator or designee will explain the patients are under no obligation to enter the Investigation and that they can withdraw at any time during the Investigation, without having to give a reason.

No clinical Investigation procedures will be conducted prior to taking consent from the participant. Consent will not denote enrolment into Investigation. A copy of the signed Informed Consent Document will be given to the participant. The original signed form will be retained at the study site and a copy placed in the medical notes.

If new safety information results in significant changes in the risk/benefit assessment, the consent form will be reviewed and updated if necessary and subjects will be re-consented as appropriate

### Informed consent and aphasia

Aphasic patients do require extra care when informed consent is being sought. All information materials will be offered in written and aural forms, and supplemented by pictorial information to ensure that they are comprehensible to all participants. Participants (and, where appropriate, their carers) will be given a minimum of one week to carefully consider the information materials before deciding about participation.

Patients who are incapable of providing informed consent (patients without capacity) will not be recruited. The vast majority of patients with post-stroke aphasia are able to consistently communicate their wishes and desires, including consent to a treatment trial, see:

Stroke and Aphasia Handbook, Parr, S., Pound, C., Byng, S. and Long, B. 2003. Connect Press, UK.

The procedures for consent outlined in the Standard Operating Procedure for informed consent form (UCL) will be adhered to. A written record of the checks made for competence to consent will be kept along with other information in each patient’s Case Report Form. The CI will also ensure that written consent is witnessed, preferably by a relation or carer of the patient, or if not, by an independent witness present at the Welcome Trust Centre for Neuroimaging, UCL (WTCN).

Written, informed consent will be obtained in all participants. We plan to follow published guidelines for addressing consent issues in aphasic patients (Penn, Frankel et al. 2009).

Only members of the clinical care team will be identifying and approaching patients.

The research team has considerable experience of working with patients with impaired speech and language abilities (there are four speech and language therapists on the team), and will take the required time and attention to ensure that the requirements and risks of the study are thoroughly understood (by both the participant and, where appropriate, a family member or carer) before consent is obtained.

### Procedure of gaining consent

Two of the research team hold weekly clinics for stroke survivors in UCLH/NHHN. They will identify potential participants for iTALKbetter and gain verbal consent to be contacted to discuss the study in more detail.

Potential participants will be telephoned again at least 1 week after initial contact, by Dr Doogan or the research assistant to answer any further questions and, if appropriate to arrange the first testing appointment at the participating site. When participants come into their first testing appointment written consent will be obtained.

At the first and subsequent appointments, potential participants will have a further opportunity to ask questions about the study.

If there is any change in capacity as indicated by a carer, the participant or a member of the research team, Dr Doogan a Clinical Psychologist, will assess this and discontinue if found to be changed.

# A.9 Schedule of assessments and interventions by visit

| **Time- point** | **iTALKbetter** |
| --- | --- |
| **T1** | - Baseline neuropsychological testing - Baseline language and cognitive assessment (including Electronic Comprehensive Aphasia Test (eCAT)) - Self-report and carer-report outcome measures |
| **T2** | - Language and cognitive assessment (including eCAT) - Self-report and carer-report outcome measures - Brain imaging |
| **T3** | - Language and cognitive assessment (including eCAT) - Self-report and carer-report outcome measures - Brain imaging   Participants are randomized at this point to one of the two versions of iTALKbetter.  Participants receive a mobile device with the DNI on it. |
| **T4** | - Language and cognitive assessment (including eCAT) - Self-report and carer-report outcome measures - Brain imaging   The mobile device with the DNI is taken away. |
| **T5** | • Language and cognitive assessment (including eCAT)  • Self-report and carer-report outcome measures. |

Some assessments may be audio and video recorded to ensure accuracy when scoring.

Some assessments may be audio and video recorded to ensure accuracy when scoring.

We will inform participants GP and therapist (if they have one) of their involvement in the study.

# A.10 Device accountability

Each DNI (software) will be given to each participant to use on hardware (a tablet computer) purchased from the grant and thus belonging to UCL. Because the software uploads data periodically to the database (on the UCL secure virtual server) we are able to tell when participants are using it. At the end of the trial block, the DNI (and tablet computer) are passed back to the research team and will be recycled for a future participant to use.

# A.11 Monitoring Plan

Case Report Forms (CRFs) will be completed by the investigator and/or his/her delegates at each timepoint. These will record any potential AEs or ADEs. These data will be collated and passed onto the IDMC for their view on safety. As each participant has several timepoints when they are not using the DNIs, we will be able to perform with-subject analyses for the IDMC to see if rates of AEs or ADEs are higher in the DNI therapy blocks.

1. Confidentiality

All data will be handled in accordance with the UK Data Protection Act 1998. The CRFs will not bear the subject’s name or other personal identifiable data. The subject’s Investigation identification number, will be used for identification. Subjects will be assigned an Investigation identification number by the study site sequentially starting with 001 upon enrolment into the study. The study site will maintain a master Subject Identification Log.

1. Record keeping and archiving

Archiving will be authorised by the Sponsor following submission of the end of study report. Chief Investigators are responsible for the secure archiving of essential Investigation documents as per their Trust policy. All essential documents will be archived for at least 5 years after completion of Investigation. Destruction of essential documents will require authorisation from the Sponsor.

The Chief Investigator will ensure there are adequate quality and number of monitoring activities conducted by the study team. This will include adherence to the protocol, procedures for consenting and ensure adequate data quality.

The Chief Investigator will inform the sponsor should he/she have concerns which have arisen from monitoring activities, and/or if there are problems with oversight/monitoring procedures.

An independent data monitoring committee (IDMC) will be set up to deal with patient safety issues related to this project. The IDMC will comprise of three independent researchers at the Institute of Neurology not linked to this project.

The data to be monitored by the IDMC will be taken from the CRFs (which will include all AEs and ADEs), as indicated above.

# A.12 Statistical Considerations

**Design**

A small, well-defined sample of patients with the potential to benefit from the DNI’s will be recruited. In both cases the main outcome measure is whether the DNI is effective at improving naming impairments. This comparison is within-subject and is achieved by comparing post-therapy measures to multiple baseline measures. A secondary question is whether the therapy can be optimized so in each case we are comparing two different versions of the therapy (a between group comparison). So, in both trials patients are randomized into one of the two types of therapy.

**Sample size calculation**

**iTalkbetter**: Average, one sample calculation, (comparison will be within group) based at three month follow up data (change in total aphasia severity score (the WAB) taken from Katz, 1997). Change in WAB after therapy block compared with no therapy block = 1.5 [SD:3.3]; Alpha error= 5%; Power = 80%. Sample Size = 30. Expect 15% drop out so target = 35 patients in total.

**Planned recruitment rate**

We will begin recruitment prior to the start of this phase and hope to have 50% of patients enrolled by the time the first patient enters the trial.

**Randomisation methods**

After iTALKbetter participants complete baseline testing (time-point 3) they will be randomised using minimisation to make sure the groups do not become unbalanced on key baseline variables. This will determine which version of the therapies participants will use and which of the word lists they are allocated (computer generated).

Participants will be randomised on the following variables: aphasia severity and time since stroke.

**Statistical analysis**

**Phase 2**

**iTALKbetter:** Data from all timepoints will be analysed using a repeated-measures ANOVA to determine whether there is an interaction between time (therapy block vs baseline) and item (trained vs untrained). The effect of the between-group factor (therapy type) is a secondary outcome measure which will also be analysed using a repeated measure ANOVA but with this extra factor added in: more formally, a three-way interaction between time (therapy block vs baseline), item (trained vs untrained) and therapy type (iTB: deterministic vs reactive).

**Both DNIs:**

Neuroimaging data, collected at three of the time points: Structural magnetic resonance imaging (MRI) data will be used to identify the lesion location and to aid co-registration of the functional MEG data. Brain structure at the three time points will be analysed using Voxel-Based Morphometry (Ashburner and Friston 2000) within the Statistical Parametric Mapping software (SPM12) to identify whether these therapies induce any structure brain changes over time. We will employ multi- parameter mapping MRI methodology for the structural brain scans (Callaghan et al, 2014). Brain imaging data (both functioning, structural and MEG) will be analysed using statistical parametric mapping 12 software (<http://fil.ion.ac.uk/sp>,). Structural MRI scans will be analysed using Voxel Based Morphometry (VBM, Ahsbrner & Friston, 2000) to test for therapy-induced changes in brain structure.

**Phase 3**

Interval behavioural data will be analysed using within-subjects, repeated measures ANOVAs (effect of DNIs over time on language and control (attention) task). Demographic data (age, time since diagnosis) and baseline psychometrics may be used as covariates in this analysis to help model between-subject variability.

# A.13 Data Management

The handling of all data on the CRFs will be the responsibility of UCL.

It will be the responsibility of the investigator to ensure the accuracy of all data entered in the CRFs. The delegation log will identify all those personnel with responsibilities for data collection and handling, including those who have access to the trial database.

We believe that these DNI’s will be easy to use and that participants will want to do their therapy as the experience of it being a digital game will be pleasurable. However, therapy is difficult and tiring so in order to minimise possible distress or frustration the research team will systematically call the participants if they are struggling to meet their targets of therapy week by week. This will enable the participant to discuss any barriers to doing the therapy and researchers can check in with participants to solve any possible but unlikely technical issues.

## *A13.1 Procedures for data review, database cleaning, and issuing and resolving data queries.*

Data entered on the CRFs will be source verified by a sponsor representative trained on the CIP and who has current GCP training. Data Clarification Forms (DCF) will be issued to the Investigator should a discrepancy be found between the source and CRF. The Investigator will be required to verify and correct all errors or provide an explanation for the discrepant data. Sponsor representatives will re-verify the corrected data and mark the clarification as resolved at the next monitoring visit.

## *A13.2 Procedures for verification, validation and securing of electronic clinical data systems*

All data from the examinations and investigations listed in Appendix A will be transferred to media provided by the sponsor and collected at the time of CRF collection.

The CI will manage and maintain the study database throughout the Investigation. At the conclusion of the Investigation, the database will then be locked and data transferred for analysis. A final copy of the database will be provided to the study site. Where data is transferred electronically, this will be in accordance with the UK Data Protection Act 1998 as well as Trust Information Governance Policy. There will be a documented record of data transfer and measures in place for the recovery of original information after transfer.

The database maintained by the CI shall be validated and secured according to the UCL standard operating procedures. Access to the data shall be limited to sponsor representatives directly involved in the collection, analysis, maintenance or safety monitoring of the data. Any study data released shall be done according to the publication policy and in accordance with the UK Data Protection Act 1998.

## *A13.3 Data retention*

Archiving will be authorised by the Sponsor following submission of the end of study report. Chief Investigators are responsible for the secure archiving of essential Investigation documents and the Investigation database as per their trust policy. All essential documents will be archived for at least 20 years after completion of Investigation. Destruction of essential documents will require authorisation from the Sponsor.

## *A13.4 Clinical quality assurance*

The Clinical Investigators will meet monthly, to discuss any issues with data quality and any concerns will be discussed with the Sponsor.

## *A13.5 Completion of Case Report Forms*

The Principal investigator will be responsible for the timing, accuracy and completeness of a CRF for each individual subject. All entries are to be made in black ink and are to be legible. All corrections made are to be completed by placing a single line through the incorrect data and the individual making the correction must initial and date the correction. Typing correction fluid must not be used. The personal data recorded on all documents will be regarded as confidential.

The Principal investigator must record the subject’s participation in this clinical investigation in the subject’s hospital notes. In addition, the Principal investigator must keep a separate list of all subjects entered into the clinical investigation showing each subject’s name, date of birth and assigned subject number (for identification purposes). A Subject Identification Log will also be provided in the Investigation Site File to record the subject’s initials and assigned subject number.

All data will be handled in accordance with the UK Data Protection Act 1998.

The CRFs will not bear the subject’s name or other personal identifiable data. The subject’s initials, date of birth and trial identification number, will be used for identification.

## *A13.6 Review and Return of Completed Documentation*

The Principal investigator will make the original Case Report Forms available to the Sponsor’s designated monitor at each visit. At the conclusion of the clinical investigation, completed Case Report Forms will be signed by the Principal investigator, collected (the original left with the Principal investigator) and a copy returned to the Sponsor.

## *A13.7 Retention of Documentation*

The Principal investigator will retain all copies of the records for a period of 20 years from the discontinuation of the clinical investigation. In all cases, the Principal investigator must contact the Sponsor prior to disposing of any records related to the clinical investigation. Included in records to be maintained are signed Clinical Investigation Plan, copies of the CRFs, signed consent forms, ethics committee approval letters, product accountability records, correspondence concerning the clinical and any other documents to identify the subjects.

In addition, if the Principal investigator moves/retires, etc., he should provide University College London with the name and address of the person who will look after and be responsible for the clinical investigation related records.

## *A13.8 Training*

During the initiation of the investigation site, the sponsor will ensure the investigators and the site study staff are trained on the device. The investigator is then responsible for ensuring that the investigation staff uses the device in the same way. All training will be documented in a Site Training Log.

The monitor will also ensure that the investigator and investigation site team have received and understood the requirements and content of:

* CIP (Clinical Investigation Plan)

* IB (Investigators Brochure)

* The informed consent forms

* CRFs (Case Report Forms)

* IFUs (Instructions For Use)

* All written clinical investigation agreements as appropriate

# A.14 Amendments to the CIP

Amendments to this CIP may be necessary to protect the safety of the patients and integrity of the data. In collaboration with the Investigator(s), the CIP amendments will be documented and submitted for ethical and regulatory approval (as required) prior to implementation. All changes will be evaluated for impact per sponsor SOPs. Amendments will be considered implemented after all ethical and regulatory approvals (as required) are received and all key sponsor and site staff has been trained. This process does not affect the individual clinician’s responsibility to take immediate action if thought necessary to protect the health and interest of individual patients.

# A.15 Deviations from clinical investigation plan

A deviation is considered a departure from the conditions and principles of GCP in connection with that Investigation; or the CIP relating to that Investigation, as amended from time to time.

The Investigator shall not deviate from this CIP except in situations that affect the subject’s rights, safety and well-being, or the scientific integrity of the clinical investigation.

## A.15.1 Procedures for recording, reporting and analysing CIP deviations

If possible, prior approval from the sponsor and REC, if appropriate, shall be obtained by the investigator. All spontaneous CIP deviations shall be recorded and reported to the sponsor as agreed. A deviation log shall be maintained by the study site. Deviations shall be reported to the REC and the regulatory authorities if required by national regulations. All deviations will be included, as required in the final study report.

**Notification requirements and time frames.**

Requests for deviations by the investigator will be responded to within [add] hours of receipt.

**Corrective and preventive actions and principal investigator disqualification criteria.**

Refer to the Monitoring Plan (as applicable) for corrective and preventative actions and principal investigator disqualification criteria.

## A.15.2 Procedure for reporting any protocol deviations

Any deviation from the protocol that has not been previously approved by the sponsor (JRO at University College London), must be reported to the sponsor within 2 working days of the deviation occurrence. Any deviations from the clinical investigation plan that are identified during routine monitoring visits will be reported to the sponsor (JRO, University College London) within 24 hours of being identified.A.16 Statements of compliance

The clinical investigation shall be conducted in accordance with the ethical principles of the Declaration of Helsinki, ISO standard 14155 and all other applicable device and UK regulations.

### The clinical investigation shall not commence recruitment until all REC, regulatory (if applicable) and local (NHS permission) is received. All additional requirements imposed by the REC or regulatory authority will be followed.

# A.16 Insurance

University College London holds insurance against claims from participants for injury caused by their participation in the clinical trial. Participants may be able to claim compensation if they can prove that UCL has been negligent. However, as this clinical trial is being carried out in a hospital, the hospital continues to have a duty of care to the participant of the clinical trial. University College London does not accept liability for any breach in the hospital’s duty of care, or any negligence on the part of hospital employees. This applies whether the hospital is an NHS Trust or otherwise.

Participants may also be able to claim compensation for injury caused by participation in this clinical trial without the need to prove negligence on the part of University College London or another party. Participants who sustain injury and wish to make a claim for compensation should do so in writing in the first instance to the Chief Investigator, who will pass the claim to the Sponsor’s Insurers, via the Sponsor’s office.

Hospitals selected to participate in this clinical trial shall provide clinical negligence insurance cover for harm caused by their employees and a copy of the relevant insurance policy or summary shall be provided to University College London, upon request.

There must also be indemnity arrangements in place, with the manufacturer, to cover the malfunction and breakdown of the device. N/A

# A.17 Adverse events, adverse device effects and device deficiencies

# a-c) Definitions

| **Term** | Definition |
| --- | --- |
| **Adverse Event (AE)** | Any untoward medical occurrence, unintended disease or injury, or untoward clinical signs (including abnormal laboratory findings) in subjects, users or other persons, whether or not related to the investigational medical device.  **Note 1:** This definition includes events related to the investigational medical device or the comparator  **Note 2:** This definition includes events related to the procedures involved  **Note 3:** For users or other persons, this definition is restricted to events related to investigational medical devices |
| **Adverse Device Effect (ADE)** | Adverse Event related to the use of an investigational device.  **Note 1:** This definition includes AEs resulting from insufficient or inadequate instructions for use, deployment, implantation, installation, or operation, or any malfunction of the investigational device  **Note 2:** This definition includes any event resulting from use error or from intentional misuse of the investigational medical device |
| **Serious Adverse Event (SAE)** | Any adverse event that:   - Led to death, - Led to serious deterioration in the health of the subject, that either resulted in - a life-threatening illness or injury, or - a permanent impairment of a body structure or a body function, or - in-patient or prolonged hospitialisation, or - medical or surgical intervention to prevent life-threatening illness or injury or permanent impairment to a body structure or a body function, - Led to foetal distress, foetal death or a congenital anomaly or birth defect |

| **Serious Adverse Device Effect (SADE)** | An ADE that has resulted in any of the consequences characteristic of an SAE |
| --- | --- |
| **Unanticipated Serious Adverse Device Effect (USADE)** | An SADE, which by its nature, incidence, severity or outcome, has not been identified in the current version of the risk analysis report. |
| **Device Deficiency (DD)** | Inadequately of a medical device with respect to its identity, quality, durability, reliability, safety or performance.  **Note 1:** this includes malfunctions, use errors, and inadequate labeling |

- An adverse event does not include:
- Medical or surgical procedures; the condition that leads to the procedure is an adverse event.
- Pre-existing disease, conditions, or laboratory abnormalities present at the start of the study that do not worsen in frequency or intensity.
- Situations where an untoward medical occurrence has not occurred (e.g., hospitalizations for cosmetic or elective surgery or social/convenience admissions);
- The disease being studied or signs/symptoms associated with the disease unless more severe than expected for the subject’s condition.

## d) Reporting requirements and timelines

AEs and ADEs are not considered reportable.

| Term | Reporter | Reported to | Reporting Timeline from awareness of the event |
| --- | --- | --- | --- |
| Adverse Event (AE) | Investigator | Sponsor | As agreed with sponsor. CI to record fully all AEs. |
| Adverse Device Effect (ADE) | Investigator | Sponsor/Manufacturer | As agreed with sponsor. CI to record fully all ADEs. |

The following events are considered reportable events in accordance with Annex 7, section 2.3.5 and Annex X, section 2.3.5 of DIRECTIVES 90/385/EEC AND 93/42/EEC respectively.

| Term | Reporter | Reported to | Reporting Timeline from awareness of the event |
| --- | --- | --- | --- |
| Serious Adverse Event (SAE)**/ Serious Adverse Device Effect (SADE) | Investigator | Sponsor | Immediately, but no more than 3 calendar days after becoming aware of the event |
|  | CI | MHRA [aic@mhra.gsi.gov.uk](mailto:aic@mhra.gsi.gov.uk) | Immediately, but not later than 2* calendar days after awareness  *For SAEs which indicate an imminent risk of death, serious injury, or serious illness and  that require prompt remedial action for other patients/subjects, users or other persons  All other events immediately but not later than 7 calendar days following date of awareness. |
|  | CI | REC | N/A |
| Unanticipated Serious Adverse Device Effect (USADE) | Investigator | Sponsor | Immediately, but no more than 3 calendar days after becoming aware of the event |
|  | CI | MHRA | Immediately, but not later than 2* calendar days after awareness  *For SAEs which indicate an imminent risk of death, serious injury, or serious illness and  that require prompt remedial action for other patients/subjects, users or other persons.  All other events immediately but not later than 7 calendar days following date of awareness. |
|  | CI | REC | Within 15 days of the chief investigator becoming aware of the event.  Only reports of related and unexpected Serious Adverse Events (SAEs) should be submitted to the REC. |

| Term | Reporter | Reported to | Reporting Timeline from awareness of the event |
| --- | --- | --- | --- |
| Device Deficiency (DD) | Investigator | Sponsor | Immediately, no more than 24 hours of becoming aware of the event |
|  | CI | MHRA | 7 calendar days  Only reportable if the event may have led to an SAE if;   - suitable action had not taken - intervention had not been made - if circumstances had been less fortunate |
| Urgent Safety Measures | CI | REC | 1. Immediately-By telephone 2. Within 3 days-Notice in writing setting out reasons for the USM and plan for further action |

** **Note** Planned hospitalisation for a pre-existing condition, or a procedure required by the CIP, without serious deterioration in health, is not considered a serious adverse event

## e) Assessments of adverse events

Each adverse event will be assessed for the following criteria:

**Severity**

| Category | Definition |
| --- | --- |
| Mild | The adverse event does not interfere with the subjects daily routine, and does not require intervention; it causes slight discomfort |
| Moderate | The adverse event interferes with some aspects of the subjects routine, or requires intervention, but is not damaging to health; it causes moderate discomfort |
| Severe | The adverse event results in alteration, discomfort or disability which is clearly damaging to health  Note: A severity rating of severe does not necessarily categorise the event as an SAE. |

### Seriousness

Seriousness as defined for an SAE in section a) above.

### Causality

The assessment of relationship of adverse events to the study procedure and the investigational device will be a clinical decision based on all available information at the time of the completion of the case report form. The following categories will be used to define the causality of the adverse event:

| Category | Definition |
| --- | --- |
| Yes | There is evidence to suggest a causal relationship, and the influence of other factors is unlikely |
| Possibly | There is some evidence to suggest a causal relationship (e.g. the event occurred within a reasonable time after procedure). However, the influence of other factors may have contributed to the event (e.g. the patient’s clinical condition, other concomitant events). |
| No | There is no evidence of any causal relationship. |

### Expectedness

| Category | Definition |
| --- | --- |
| *Expected* | An adverse event that is consistent with the information about the device listed in the Investigator Brochure or clearly defined in this CIP. |
| *Unexpected* | An adverse event that is not consistent with the information about the device listed in the Investigator Brochure |

The reference document to be used to assess expectedness against the intervention is the IB. The CIP will be used as the reference document to assess disease related and/or procedural expected events.

## f) Procedures for recording and reporting Adverse Events and Device Deficiencies

### Investigator responsibilities:

All adverse events and SAEs will be recorded in the medical records and CRF following consent.

All serious adverse events will need to be reported to the sponsor on a SAE form (using MEDDEV form 2.7/3) unless stated in the CIP that some expected SAEs will not be reported to the sponsor, with a justification as to why they will not be reported.

For patients on the control arm of an Investigation, SAEs may not have to be reported to the sponsor but will be recorded in the CRF and medical records.

The Chief or Principal Investigator will complete the serious adverse event form and the form will be emailed to the sponsor UCL, within 3 working day of his/her becoming aware of the event. The Chief or Principal Investigator will respond to any SAE queries raised by the sponsor as soon as possible.

The Investigator will report to the MHRA and REC (as applicable) all reportable events within the specified timeframes as per section d above.

***All deaths will be reported to the sponsor irrespective of whether the death is related to disease progression, the device, or an unrelated event.***

All serious adverse events will be recorded in the medical records and the CRF, and the sponsor’s AE log.

All SAEs (except those specified in section d as not requiring reporting to the Sponsor) must be recorded on a serious adverse event (SAE) form. The CI/PI or designated individual will complete the sponsor’s SAE form and the form will be preferably emailed to the Sponsor within 5 working days of becoming aware of the event. The Chief or Principal Investigator will respond to any SAE queries raised by the sponsor as soon as possible.

Where the event is unexpected and thought to be related to the intervention, this must be reported by the Investigator to the Health Research Authority within 15 day.

All SAEs and UADEs should be reported to the following; [insert REC and MHRA]

## Reporting of all Adverse Events and Device Deficiencies: Investigator and Sponsor responsibilities

Investigator responsibilities shall be as per section d). The sponsor shall keep detailed records of all adverse events and device deficiencies relating to the clinical Investigation, which are reported to them by the Investigation investigators. The sponsor shall ensure that all relevant information about a reportable event, which occurs during the course of this clinical Investigation in the United Kingdom, is reported as soon as possible to the MHRA, and the relevant ethics committees per their reporting requirements and according to the timelines in section d. Any additional relevant information should be sent within the same time frame as the initial report. The CI is responsible for informing the appropriate regulatory authorities, ethics committees and other investigators of any reportable events that have occurred with the study device in any clinical investigation according to the guidelines set forth by either the REC of record or regulatory authority in the country where the clinical investigation is taking place.

### Progress reports

Progress reports will be submitted to the REC as per the REC requirements. The chief investigator will prepare the annual progress reports.

**Foreseeable adverse events and anticipated adverse device effects**

### Anticipated adverse events and adverse device effects (ADEs), together with their likely incidence, mitigation or treatment.

**Adverse events**

Patients with stroke are at risk for both further strokes and morbidity and mortality associated with co-existing cardiovascular disease. Some progression in dementia is expected however significant changes in the disease profile may also occur.

1. Further strokes.
2. Myocardial infarction.
3. Angina.
4. Peripheral vascular disease.
5. Significant progression of dementia

If any brain abnormality is found on the MRI scans during the study the following procedure will be adhered to. All abnormal structural scans will be shown to a Consultant Neuroradiologist from the National Hospital for Neurology and Neurosurgery, as soon as possible.

Prof Alex Leff, Consultant Neurologist will have responsibility for reporting the finding to the participant’s GP and discussing it with the participant as soon as possible.

A record of events will be kept in the research log however they will not be reported to REC and MHRA as they are anticipated events that patients are at risk of due to the nature of the underlying condition being studied. The records of these events will be discussed by the IDMC.

**Adverse device effects**

Regarding ADEs the following symptoms are the commonest ones associated with computer use according to the American Optometric Association: eyestrain, headaches, blurred vision and neck or shoulder pain. These occur in up to 14% of subjects who use computers regularly at work. At each time point we will document the presence or absence of these symptoms and record them in the CRF. This will be passed onto the IDMC. Regarding mitigation, we will advise all of our participants to moderate their use of the DNIs according to these symptoms; that is, to take a rest if they experience them.

# A.18 Oversight Committees

**Trial Management Group (TMG)**

The TMG will include the Chief and Principal Investigators and experts from relevant specialties.  The TMG will be responsible for overseeing the trial.  The group will meet at least twice per year and all members will sign a TMG charter.  The TMG will review substantial amendments to the protocol prior to submission to the REC and MHRA.

**Trial Steering Committee (TSC )**

As this is not a multicenter trial, the TMG and TSC are one and the same.

**Independent Data Monitoring Committee (IDMC)**

The role of the IDMC is to provide independent advice on data and safety aspects of the trial.  Meetings of the Committee will be held annually to review the safety data generated by the study or as necessary to address any issues.  Information about adverse events and device effects will be gathered at each of the clinical trial time-points per patient. If the IDMC deems any adverse event or device effect is excessively high (i.e statistically and clinically significant) then they can recommend premature closure of the trial.

The IDMC is advisory to the TSC and can recommend premature closure of the trial to the TSC. All IDMC members will sign an IDMC charter.

# A.19 Vulnerable population

1. **Description of the vulnerable population.**

**Stroke**

Some stroke survivors may be considered vulnerable as they may have significant communication and cognitive difficulties. However, as part of this clinical trial we will not be recruiting anyone with significant cognitive impairment and therefore on an individual basis may not be considered as vulnerable.

Some support with travelling to appointments by family members which we will encourage.

1. **Description of the specific informed consent process.**

**iTALKbetter**

However due to the difficulties with communication that aphasic patients do have they require extra care when informed consent is being sought. All information materials will be offered in written and aural forms, and supplemented by pictorial information to ensure that they are comprehensible to all participants. Participants (and, where appropriate, their carers) will be given a minimum of one week to carefully consider the information materials before deciding about participation.

Patients who are incapable of providing informed consent (patients without capacity) will not be recruited. The vast majority of patients with post-stroke aphasia are able to consistently communicate their wishes and desires, including consent to a treatment trial, see:

Stroke and Aphasia Handbook, Parr, S., Pound, C., Byng, S. and Long, B. 2003. Connect Press, UK.

1. **Description of the EC's specific responsibility. N/A**

**d) Description of what medical care, if any, will be provided for subjects after the clinical investigation has been completed.**

Participants will receive free access to the DNIs if they wish after phase 2 in phase 3.

# A.20 Suspension or premature termination of the clinical investigation

Both the Sponsor and the Principal investigator reserve the right to terminate the clinical investigation at any time. Should this be necessary, the procedures will be arranged on an individual basis after review and consultation by both parties. In terminating the clinical investigation, the JRO at University College London and the Principal investigator will assure that adequate consideration is given to the protection of the subject’s interests.

## A20.1 Subject Withdrawals and Discontinuation

A participant may be withdrawn from the trial whenever continued participation is no longer in the participant’s best interests, but the reasons for doing so must be recorded. Reasons for discontinuing the trial may include:

- Subject’s rescission of consent.
- Any unexpected adverse device effect which is, in the opinion of the Principal investigator, related to the device and will endanger the wellbeing of the subject if the treatment is continued.
- The development of any undercurrent illness (es), infection or condition(s) that might interfere with the Clinical Investigation Plan.
- Any problem deemed by the Principal investigator and/or the JRO at University College London to be sufficient to cause discontinuation.
- Disease progression whilst in the study
- Intercurrent illness
- Patients withdrawing consent
- Persistent non-compliance to protocol requirements.

All subjects discontinued from the clinical investigation due to an unexpected adverse device effect, directly related to the clinical investigation, will be treated until the effect resolves. The Principal investigator will clearly document the date and reason(s) for subject withdrawal in his/her CRF and the monitor must be notified.

Subjects who are withdrawn will not be replaced if they have received the investigational device. If possible, any procedures or assessments planned for the subject on withdrawal from the clinical investigation should be performed when intention to withdraw the subject is announced.

Subjects who are withdrawn prior to receiving investigational device will be replaced.

# A.21 Definition of End of Trial

The expected duration of the trial is 3 years from recruitment of the first participant. The end of the trial (phase 2) is the date of the last visit of the last participant.

# A.22 Publication policy

Yes we plan to publish the findings.

As we are NIHR funded the NIHR need to be sent a draft at the time of submission. The rules are here:

“The NIHR actively encourages the dissemination of research. Investigators and their research teams funded under

an NIHR programme should provide advance notice of research outputs. A copy of the proposed publication

should be sent to the funding programme at the same time as submission for publication or at least 28 days before

the date intended for publication whichever is earlier. Please note this also applies to press releases to be issued by your host institution, e.g. university, NHS Trust or hospital. (Please see 4.1 on Press Releases)”

<https://www.nihr.ac.uk/funding-and-support/documents/funding-for-research-studies/manage-my-study/outputs-guidance-notes-2.pdf> (page 2).

All proposed publications will be discussed with and reviewed by the Sponsor prior to publishing other than those presented at scientific forums/meetings. Please refer to UCL publication policy.

The results of the study will be reported in the following ways: peer reviewed scientific journals; internal report; conference presentation; written feedback to research participants.

Please refer to UCL publication policy.

**Intellectual Property**

All background intellectual property rights (including licences) and know-how used in connection with the study shall remain the property of the party introducing the same and the exercise of such rights for purposes of the study shall not infringe any third party’s rights.

All intellectual property rights and know-how in the protocol and in the results arising directly from the study, but excluding all improvements thereto or clinical procedures developed or used by each participating site, shall belong to UCLH. Each participating site agrees that by giving approval to conduct the study at its respective site, it is also agreeing to effectively assign all such intellectual property rights (“IPR”) to UCL and to disclose all such know-how to UCL.

Each participating site agrees to, at the request and expense of UCL execute all such documents and do all acts necessary to fully vest the IPR in UCL.

Nothing in this section shall be construed so as to prevent or hinder the participating site from using know-how gained during the performance of the study in the furtherance of its normal activities of providing or commissioning clinical services, teaching and research to the extent that such use does not result in the disclosure or misuse of confidential information or the infringement of an intellectual property right of UCL. This does not permit the disclosure of any of the results of the study, all of which remain confidential.

# A.23 Bibliography

List of the literature and data that are relevant to the trial, and that provide background for the trial. Please ensure the text contains appropriate cross references to this list

Ashburner, J. and K.J. Friston (2000). “Voxel-based morphometry-the methods”. Neuroimage, **11** (6 Pt 1): 805-821.

Bhogal, S.K., R. Teasell, and M. Speechley (2003). “Intensity of aphasia therapy, impact on recovery”.

Stroke, **34**(4): 987-93.

Brady, M.C., H. Kelly, J. Godwin, and P. Enderby (2013). “Speech and language therapy for aphasia following stroke”. Cochrane Database of Systematic Reviews, **12**(5).

Callaghan, J., J. Wells, S. Richardson, H. Holmes, Y. Yichao, S. Walker-Samuel, B. Siow, and M.F. Lythgoe (2014). “Is Your System Calibrated? MRI Gradient System Calibration for Pre-Clinical, High-Resolution Imaging”. Plos one: https://doi.org/10.1371/journal.pone.0096568.

Code, C. and C. Heron (2003). “Services for aphasia, other acquired adult neurogenic communication and swallowing disorders in the United Kingdom, 2000.” Disability Rehabilitation **25**(21): 1231- 1237.

Clare L., B.A. Wilson, G. Carter, I. Roth, and J.R. Hodges (2002). “Relearning face-name associations in early Alzheimer’s disease”. Neuropsychology, **16**(4): 538-47.

Craig P., P. Dieppe, S. Macintyre, S. Michie, I. Nazareth, and M. Petticrew (2008). “Developing and evaluating complex interventions: the new Medical Research Council guidance”. British Medical Journal, **33**(7): a 1655.

Moss A., and M. Nicholas (2006). “Langauge Rehabilitation in chronic aphasia and time postonset”.

Stroke, **37**(12): 3043-3051.

Palmer, R., Enderby, P., Cooper, C., Latimer, N., Julious, S., Paterson, G. & Delaney, A. (2012). Computer therapy compared with usual care for people with long-standing aphasia poststroke: a pilot randomized controlled trial. *Stroke*, *43*(7), 1904-1911.

Prince, M., M. Knapp, M. Guerchet, M. P. McCrone, M. Prina, A. Comas-Herrea, R. Wittenberg et al. (2014). “Dementia UK: Update”. London: Alzheimer’s Society.

Swinburn, K., G. Porter, and D. Howard (2004). “Comprenhensive Aphasia Test”. Psychology Press.

Werner, P. (2004). “Beliefs about memory problems and help seeking in elderly persons”. Clinical Gerontologist, **27**(4): 19-30.

| ***iTALKbetter*** | ***Baseline***  ***1*** | ***Baseline 2*** | ***Baseline 3*** | | *Treatment* | | **Time-point 4** | **Final Time-point** | |
| --- | --- | --- | --- | --- | --- | --- | --- | --- | --- |
| *Visit #* | *1* | *2* | | *3* | |  | *4* | *5* |  |
|  | *Week 0* | *Week 6* | | *Week 12* | | *6 weeks duration* | *Week 18* | *Week 30* |  |
| ***Informed Consent*** | *X* |  | |  | |  |  |  |  |
| ***Medical History*** | *X* |  | |  | |  |  |  |  |
| ***Baseline neuropsych tests*** | *X* | *X* | | *X* | | *X* | *X* | *X* |  |
| ***Language Tests*** | *X* | *X* | | *X* | | *X* | *X* | *X* |  |
| ***Proms*** | *X* | *X* | | *X* | | *X* | *X* | *X* |  |
| ***Randomisation ^d^*** |  |  | | ***X*** | |  |  |  |  |
| ***Brain Scans*** |  | *X* | | *X* | |  | *X* |  |  |
| ***Device/Treatment*** |  |  | |  | | *X* |  |  |  |
| ***Adverse Events review (CRF)*** | *X* | *X* | | *X* | | *X* | *X* | *X* |  |
| ***Concomitant Medication review*** | *X* |  | |  | |  |  |  |  |
|  |  |  | |  | |  |  |  |  |
